# Spatio-temporal metabolic rewiring in the brain of TgF344-AD rat model of Alzheimer’s disease

**DOI:** 10.1038/s41598-022-20962-6

**Published:** 2022-10-10

**Authors:** Emma Muñoz-Moreno, Rui Vasco Simões, Raúl Tudela, Xavier López-Gil, Guadalupe Soria

**Affiliations:** 1 Magnetic Resonance Core Facility, Institut d’Investigacions Biomeiques August Pi i Sunyer (IDIBAPS), Barcelona, Spain; 2 Institute for Research & Innovation in Health (i3S), University of Porto, Porto, Portugal; 3 Group of Biomedical Imaging, University of Barcelona, Barcelona, Spain; 4 Laboratory of Surgical Neuroanatomy, Faculty of Medicine and Health Sciences, Institute of Neurosciences, University of Barcelona, Barcelona, Spain; 5 CIBER de Bioingeniería, Biomateriales y Nanomedicina, Instituto de Salud Carlos III, Barcelona, Spain

**Keywords:** Neuroscience, Alzheimer's disease

## Abstract

Brain damage associated with Alzheimer's disease (AD) occurs even decades before the symptomatic onset, raising the need to investigate its progression from prodromal stages. In this context, animal models that progressively display AD pathological hallmarks (e.g. TgF344-AD) become crucial. Translational technologies, such as magnetic resonance spectroscopy (MRS), enable the longitudinal metabolic characterization of this disease. However, an integrative approach is required to unravel the complex metabolic changes underlying AD progression, from early to advanced stages. TgF344-AD and wild-type (WT) rats were studied in vivo on a 7 Tesla MRI scanner, for longitudinal quantitative assessment of brain metabolic profile changes using MRS. Disease progression was investigated at 4 time points, from 9 to 18 months of age, and in 4 regions: cortex, hippocampus, striatum, and thalamus. Compared to WT, TgF344-AD rats replicated common findings in AD patients, including decreased N-acetylaspartate in the cortex, hippocampus and thalamus, and decreased glutamate in the thalamus and striatum. Different longitudinal evolution of metabolic concentration was observed between TgF344-AD and WT groups. Namely, age-dependent trajectories differed between groups for creatine in the cortex and thalamus and for taurine in cortex, with significant decreases in Tg344-AD animals; whereas myo-inositol in the thalamus and striatum showed greater increase along time in the WT group. Additional analysis revealed divergent intra- and inter-regional metabolic coupling in each group. Thus, in cortex, strong couplings of N-acetylaspartate and creatine with myo-inositol in WT, but with taurine in TgF344-AD rats were observed; whereas in the hippocampus, myo-inositol, taurine and choline compounds levels were highly correlated in WT but not in TgF344-AD animals. Furthermore, specific cortex-hippocampus-striatum metabolic crosstalks were found for taurine levels in the WT group but for myo-inositol levels in the TgF344-AD rats. With a systems biology perspective of metabolic changes in AD pathology, our results shed light into the complex spatio-temporal metabolic rewiring in this disease, reported here for the first time. Age- and tissue-dependent imbalances between myo-inositol, taurine and other metabolites, such as creatine, unveil their role in disease progression, while pointing to the inadequacy of the latter as an internal reference for quantification.

Alzheimer’s disease (AD) represents the most common cause of dementia and a major healthcare burden. Compelling evidence demonstrates AD as a continuous, progressive disease, with brain damage occurring decades before the symptomatic onset^1,2^. Hence, the early characterization and understanding of this pathological cascade is of great interest. Particularly, brain metabolic alterations associated with AD have been investigated as potential disease biomarkers in patients, mostly based on magntetic resonance spectroscopy (MRS)^3,4^. This non-invasive technique allows the in-vivo assessment of brain metabolic profiles and has been applied to follow-up at-risk or preclinical AD subjects^5,6^, as well as to monitorize the effects of therapeutic interventions^7,8^. The most consistent MRS findings in AD patients have been decreased levels of N-acetylaspartate (NAA) in different brain regions, such as the posterior cingulate cortex and hippocampus, and increased myo-inositol-to-creatine ratio (Ins/Cr) in the posterior cingulate cortex and parietal grey matter^4^. Decreased levels of glutamine and glutamate (Glx pool) and increased Ins/NAA have also been reported^4^. Decreased NAA/Cr and increased Ins/Cr have been detected in earlier phases of the disease, as well as in mild cognitive impairment (MCI) patients and healthy at-risk subjects^6^. Accordingly, decreased NAA/Ins and NAA/Cr have been associated with higher *tau* protein burden, and increased Ins/Cr and decreased NAA/Ins with higher deposition of amyloid-β (Aβ) plaques^5^. However, exploring the true potential of MRS-based biomarkers requires a thorough characterization of the metabolic pattern changes occurring at different disease stages and brain regions. For this, animal models of AD represent a very convenient approach to study disease progression, particularly at its earliest stages^9,10^.

Animal models are suitable for longitudinal studies of AD, since the whole lifespan can be analysed significantly faster than in humans and under controlled conditions^11,12^. Importantly, these models should mimic as close as possible the metabolic changes observed in AD patients and should be studied with the same technologies, to ensure the research is clinically translatable^10^. So far, brain metabolic changes have been mainly evaluated in mouse models of the disease^13–17^. As in humans, the most common finding is decreased NAA or NAA/Cr in cortex or hippocampus. Decreased levels of Glu or Glu/Cr have also been reported^13,18–20^. However, the overall results with mouse AD models are less consistent compared to human cohorts, arguably due to differences in the pathological changes replicated by each model and the different regions and disease stages evaluated by MRS. For instance, there is no consensus about Ins or Ins/Cr changes: while some studies reported increases^21,22^, others reported no alterations^19,20,23^. Moreover, it has been hypothesised that the increased taurine (Tau) described in some of these models might translate to the increased myo-inositol levels observed in humans^18^.

Despite more frequent use of mouse AD models, rat models present several advantages: closer to humans in evolution, and therefore more similar in physiology, morphology and genetics, rats allow the use of more complex behavioural tests than mice; and their larger size facilitates the use of more robust neuroimaging approaches^24,25^. Specifically, the ability to select larger regions of interest than in the mouse brain reduces partial volume effects, thereby providing higher quality MRS data. So far, MRS findings have mainly been reported in two rat models of AD: the McGill-R-Thy1-APP^26^ and the TgF344-AD^27^. A longitudinal MRS study conducted in the McGill-R-Thy1-APP rat model reported NAA decrease at 9 months of age in the hippocampus and frontal cortex, while an increase was observed in the cortex at 12 months; in the hippocampus Ins was decreased in young animals, transiently increased at 9 months and similar to controls at 12 months; higher taurine levels and lower Glu and GABA were also reported in these two regions at different ages^28^. While the McGill-R-Thy1-APP model shows Aβ plaques already at 6 months of age, it does not replicate other aspects of the disease, such as neurofibrillary tangles or widespread cell death^26^. Indeed, neurodegeneration was only reported at 18 months of age in this model^9^.

The TgF344-AD transgenic rat model manifests age-dependent cerebral amyloidosis, tauopathy, gliosis, apoptotic loss of neurons, and cognitive impairment^27^. The clearest pathological hallmarks were identified from 16 months on, with neuronal loss, tauopathy and Aβ plaques deposition clearly observed in the cortex, hippocampus and striatum^27^. Since the emergence of TgF344-AD rats, many other alterations have been reported in this model affecting neurotransmission, neuronal connectivity, neurovascular function and cognition among others^12,29–31^. Two recent studies have evaluated brain metabolites in TgF344-AD rats: Chaney et al.^32^ reported decreased NAA levels in the hippocampus and increased Tau in the thalamus of 18 months old TgF344-AD rats, together with assessment of neuroinflammation, the acetylcholine system, Aβ plaque deposition and *tau* protein aggregates; while^33^ also reported a decrease in hippocampal NAA starting at 10 months of age together with decreases of taurine and creatine and increases in choline.

In our work, we aim to go beyond specific brain region analyses and provide a more global perspective including the cross-talks of metabolic imbalances across brain regions. To do this, we propose a framework for analysing the spatio-temporal brain metabolic profile changes associated with AD progression, using the TgF344-AD rat model. Specifically, we carried out a longitudinal analysis with four time points (9, 12, 15 and 18 months of age), capturing from prodromal to advanced disease stages in four brain areas: cingulate cortex, hippocampus, thalamus and striatum. Amyloid deposition has been first observed in cortex and later detected in hippocampus and striatum in this transgenic model^27^ and in AD patients^34^. While the involvement of hippocampus and cortex in AD has been extensively investigated, the role of striatum has been recently highlighted: at early stages of the disease, striatal Aβ plaques are characteristic of familial AD but not observed in the sporadic subtype, where only appear at advanced stages^35^. Indeed, striatal Aβ measurements were better correlated with *tau* burden and memory scores than the neocortical cortex, suggesting that striatal amyloid deposition might predict disease severity in the preclinical stage of autosomal dominant AD^36^ Regarding the thalamus, its role in the disease has drawn attention in the last years, pointing to structural alterations in this region appearing at the earliest stages of the disease and involved in cognitive decline and memory loss^37,38^. Therefore, the choice of these four areas allows us to evaluate the topographical and temporal pattern of metabolic alterations.

Thus, we evaluated the ability of the TgF344-AD model to reproduce the brain MRS changes mainly observed in AD patients. Moreover, we provide new insights into the underlying metabolic processes associated with the pathology by analysing their inter- and intra-regional couplings. To ensure the reliability of the results, we propose a quantitative approach with rigorous quality control, from data acquisition to processing and analysis.

## Methods

### Subjects

The experiments were carried out in a cohort of 18 male rats including: 9 TgF344-AD rats^27^ and 9 wild-type (WT) Fisher littermate rats, that were evaluated by MRS every 3 months, from 9 to 18 months of age. Rats were housed in cages under controlled temperature (21 ± 1 °C) and humidity (55 ± 10%) with a 12 h light/12 h dark cycle (light between 8:00 AM and 8:00 PM). Food and water were available ad libitum during all experiments. Due to complexity of the experimental design and limited MRI access, not all the animals could be scanned at the 4 time points. Table 1 compiles the group sizes and median age at each evaluated time point.

**Table 1:** Longitudinal MRI/MRS experiment. Sample sizes and age.

Time point	Wild-type		TgF344-AD	
	N	Age (months)	N	Age (months)
1	9	8.43 ± 0.50	8	8.93 ± 0.26
2	9	11.47 ± 0.27	9	11.53 ± 0.07
3	9	15.03 ± 0.30	9	15.07 ± 0.07
4	8	17.87 ± 0.53	6	18.28 ± 0.68

Number of MRS datasets acquired per group, time point, and age (median ± interquartile range).

### MRI/MRS acquisition

Magnetic resonance imaging (MRI) experiments were conducted on a 7.0Tesla BioSpec 70/30 horizontal animal scanner (Bruker BioSpin, Ettlingen, Germany), equipped with an actively shielded gradient system (400 mT/m, 12 cm inner diameter) and a 4-channel phased-array receiver surface RF coil, for the rat brain. Animals were placed in supine position in a Plexiglas holder with a nose cone for administering anaesthetic gases (1.5% isoflurane in a mixture of 30% O_2_ and 70% N_2_O) and were fixed using a tooth bar, ear bars, and adhesive tape. Animal physiology, i.e. rectal temperature and breathing, were constantly monitored during acquisition (control/gating module and PC-SAM 32 v8.02, Small Animal Instrument Inc., Stony Brook, NY, USA), and kept between 34 and 38 °C and 60–80 breaths per minute, respectively. All acquisitions were performed during the light period.

3D-localizer scans were used to ensure the head position at the isocenter of the magnet, and 1st and 2nd order shimming performed with MapShim, considering an ellipsoid volume adjusted to fit the brain. A T2-weighted RARE (rapid acquisition with relaxation enhancement) sequence was used to image the whole brain, with an effective echo time (TE) of 35.3 ms, repetition time (TR) 6000 ms and RARE factor 8. Matrix size was 256 × 256 with an in-plane voxel size of 0.12 × 0.12 mm^2^, 40 slices, slice thickness 0.8 mm, resulting in a field of view (FOV) of 30 × 30 × 32 mm^3^.

**Figure 1:**
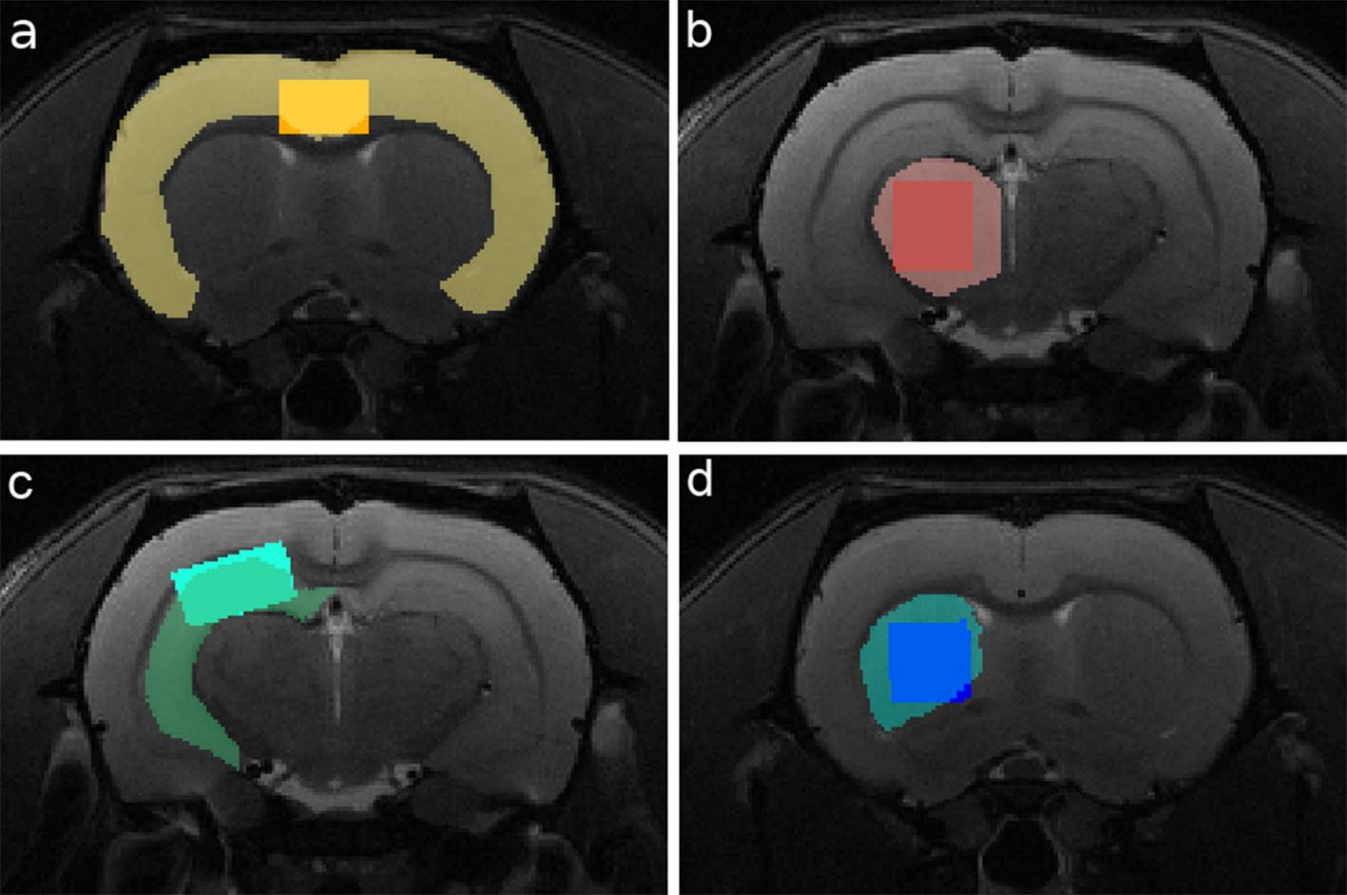
MRS voxel location. Position and size of the four MRS voxels in representative coronal slices of the same animal, overlaid in the respective T2-weighted RARE images and the segmentation of the region of interest: (**a**) cortex; (**b**) right thalamus; (**c**) right hippocampus and (**d**) right striatum.

Reference T2-weighted images were acquired in axial, coronal and sagittal orientations to accurately position the four MRS voxels: hippocampus, 11.88 µL; thalamus, 15.40 µL; striatum, 15.75 µL; and cingulate cortex, 12.60 µL (Fig. 1). For each voxel, MRS was acquired with PRESS localization (TE = 12 ms, TR = 5000 ms) using the following protocol: first, local 1st and 2nd order shimming (MapShim) performed in the voxel followed by a reference water spectrum acquired with 8 repetitions, to ensure a peak full width at half-maximum (FWHM) ≤ 12 Hz; then, MRS was acquired with 256 repetitions and VAPOR water suppression, adjusted to keep the water peak amplitude 30–50% higher than the upfield metabolite peaks.

### MRS quantification and quality control

MRS data sets were analysed with LCModel^39^. Thus, brain metabolites were detected and quantified by linearly fitting each spectrum with a simulated basis set including 19 metabolites, as well as macromolecules. Absolute concentrations were estimated based on the respective reference water spectrum. To ensure reliability of the data, spectra with FWHM > 0.06 ppm or signal-to-noise ratio (SNR) < = 10 were discarded, as well as estimated metabolite concentrations with a relative Cràmer Raw Lower Bound (%SD) > 15. Metabolite concentrations were further corrected for partial volume effects, accounting for the relative contributions of white matter (WM), grey matter (GM) and corticospinal fluid (CSF) present in the voxel^39^. The latter were estimated based on automatic tissue segmentation performed in the T2-weighted RARE dataset. First, ANTs elastic registration^40^ was used to translate the tissue probability maps (TPM) for WM, GM and CSF provided in^41^ to the image acquired from each subject and time point. Then, tissue segmentation was performed by the unified segmentation algorithm implemented in SPM^42^, using the registered TPM as a priori probability maps. The area covered by each of the MRS voxels was identified over the images, and the percentage of WM, GM and CSF was computed. Besides, a rat brain atlas^43^ was used for automatic parcellation of the brain in anatomical regions as described in^44^ and the percentage of the MRS voxel corresponding to the region of interest was assessed. These percentages were also considered as quality criteria about the accuracy of voxel location.

To account for potential confounders related to physiological conditions, besides the rectal temperature and breathing measured during acquisition, brain temperature was additionally estimated from the acquired spectra according to^45^ based on the chemical-shift of NAA (f_NAA_) relative to the residual water peak (f_H2O_) as follows: *T*^*b *^= 36 + 106.38 (f_H2O _-f_NAA _-2.6759).

### Statistical analysis

#### Linear mixed effect model

Due to acquisition or experimental issues and after quality control and outlier removal in each group (using 1.5 interquartile range as criteria), we could have missing values for specific metabolite concentration at one or more of the evaluated time points and regions. To deal with this and also account for heterogeneities in basal concentrations, longitudinal analysis was performed by fitting a linear mixed effect (LME) model to each time-course. LME models take into account both fixed effects, common to the entire population (age and group), and random effects, i.e. subject-specific parameters to model the deviation of each subject from the population^46^. Each metabolite concentration was modelled as a function of group and age, and their interaction. Since not all the metabolites showed a clear linear temporal evolution, we considered a quadratic dependence with age according to Eq. (1):

1 $$c_{mb,s} = \beta_{0} + \beta_{1} \;genotype + \beta_{2} \;age^{2} + \beta_{3} \;genotype\;age^{2} + \beta_{4,s} \;s + \xi$$

where *c*_*mb,s*_ is the concentration of the metabolite *mb* in subject *s* at a given *age*; *β*_*0*_ is the global intercept; *β*_*1,*_* β*_*2,*_* β*_*3*_ are the fixed-effect parameters, assessing the influence of group, age, and interaction respectively; *β*_*4,s*_ is the subject-specific correction and ξ is the regression error term.

Based on^47^, the minimum sample size for longitudinal analysis of each metabolite was estimated to be 40, that is, if there were less than 40 concentration values estimated with enough quality for a given metabolite at a specific region, this metabolite was not considered for the analysis.

The significance of group, age or their interaction, was evaluated from the LME model fitting. In case of significant interaction between age and group (*p* < 0.05), the group-specific age effect was evaluated by the model fitted separately to each of the groups. In addition, for those metabolites with significant group or interaction effects (*p* < 0.05), the differences at each of the evaluated time points were assessed with Kruskal–Wallis statistics. Effect size of each parameter in the LME model and Kruskal–Wallis were estimated by Cohen’s f^2^ and η^2^, respectively. This specific time point comparisons were only performed if there were a minimum of 6 samples per group.

#### Intra- and inter-regional correlations

Intra- and inter-regional associations between metabolites were evaluated with the Spearman’s correlation, for the WT and the TgF344-AD group. To discard spurious correlations, only highly significant correlations (*p* < 0.001) were considered.

### Ethics approval and consent to participate

All animal work was performed following the local legislation (Decret 214/1997 of July 20th by the Departament d’Agricultura, Ramaderia i Pesca de la Generalitat de Catalunya) under the approval of the Experimental Animal Ethical Committee of the University of Barcelona, CEEA (committee’s reference number 10724), and in compliance with European legislation.

## Results

### Quality control

After the initial quality control assessment, 24 out of 252 spectra (9%) were discarded according to the FWHM and SNR selection criteria, including different brain regions: cortex, 8 (4 TgF344-AD, 4 WT); hippocampus, 2 (1 TgF344-AD, 1 WT); striatum, 1 (1 TgF334-AD); and thalamus, 13 (10 TgF344-AD, 3 WT). For more details about the distribution of the discarded spectra at specific timepoints, see Supplementary Table 1. In addition, metabolite concentrations not estimated with enough reliability (%SD > 15) were also discarded. After quality control, outlier detection and sample size criteria, the metabolites included in the study were: creatine (Cr), total creatine (tCr), glutamate (Glu), glutamine (Gln), glutamate and glutamine pool (Glx), total choline compounds (tCho), glutathione (GSH), myo-inositol (Ins), myo-inositol and glycine pool (Ins+Gly), N-acetylaspartate (NAA), total NAA (tNAA) including NAA and N-acetylaspartylglutamate (NAAG), in all regions; and phosphocreatine (PCr) and taurine (Tau), in all regions but thalamus.

**Figure 2:**
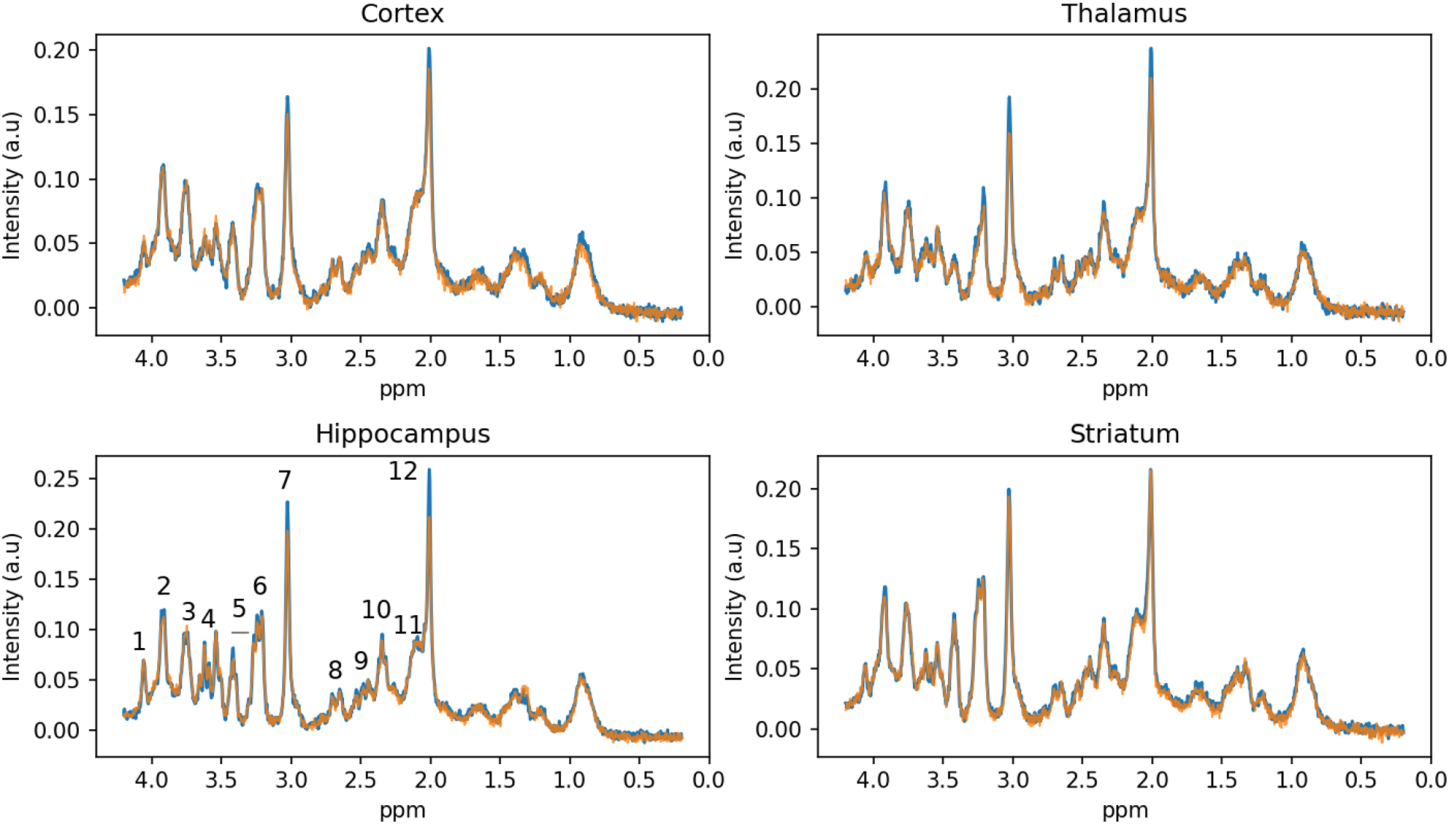
Regional metabolic profiles in aged rats. Average MRS profiles for each of the four brain regions evaluated in the WT (blue) and TgF344-AD (orange) cohorts at 18 months of age. Peaks corresponding to the main metabolites are indicated in the hippocampus profile: 1, myo-inositol (Ins); 2 and 7, total creatine (tCr); 3 and 11, glutamate + glutamine pool (Glx); 4, myo-inositol and glycine (Ins+Gly); 8 and 12, total N-acetylaspartate (tNAA); 5, taurine (Tau); 6, choline compounds (tCho); 9, glutamine (Gln); 10, glutamate (Glu). For quantitative information of median and interquartile range of the metabolite peaks, see Supplementary Table 2.

As an example of the quality of the obtained metabolic profiles, Fig. 2 shows the average profile for each group at 18 months of age in the four evaluated regions.

Atlas-based parcellation of each brain was used to assess the percentage of the region of interest included in the MRS voxel. No differences between TgF344-AD and WT animals were observed, as shown in Supplementary Table 3. Rectal and estimated brain temperature are also reported in Supplementary Table 3, showing no differences between transgenic and WT animals in hippocampus or striatum (acquired first), while slightly lower temperatures were detected in the striatum and cortex (acquired later) of the transgenic group.

### Group and age effects in longitudinal metabolite profiles

**Figure 3:**
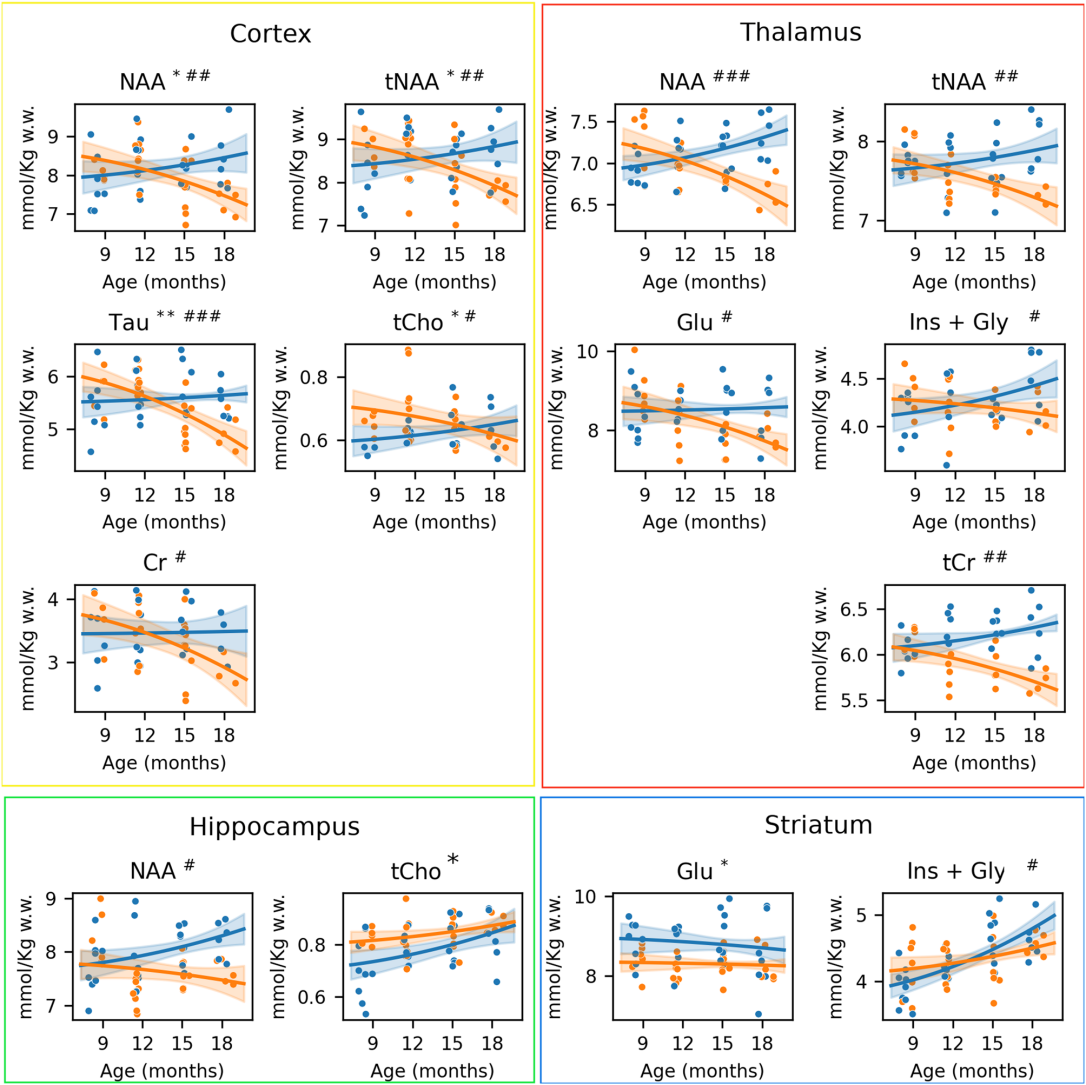
Longitudinal analysis of metabolite concentrations. Time-course metabolic changes in TgF344-AD (orange) and WT (blue) animals displaying only metabolites/regions with significant effect of group or group-age interaction in the LME model. Solid lines represent the LME model fitting, each dot corresponding to one sample (subject and time point). *: *p*-value of the group effect < 0.05; **: *p*-value of the group effect < 0.01; #: *p*-value of the group-age interaction < 0.05; ## *p*-value of the group-age interaction < 0.01; ###: *p*-value of the group-age interaction < 0.001.

Figure 3 displays the time-course of metabolic concentrations where a significant group or group-age interaction was observed. *p*-values and Cohen’s f^2^ effect sizes of the LME model for each factor and their interaction are compiled in Supplementary Table 4, including not only the metabolites showing group or interaction effects but also the metabolites that were affected by age. If a significant interaction between age and group was observed, LME model was fitted groupwise. Supplementary Table 5 shows the significance of aging effect in each group.

The cortex and thalamus were the regions where more metabolites were found to be altered. Specifically, N-acetylaspartate (NAA and tNAA) showed a significant interaction between age and genotype (NAA: p_inter, ctx_ = 0.0027, p_inter, tha_ = 6.86 10^−6^; tNAA: p_inter, ctx_ = 0.0049, p_inter, tha_ = 0.0036) and significantly decreased with age in the transgenic group (NAA: p_age, ctx_ = 0.0024, p_age, tha_ = 0.0006; tNAA: p_age, ctx_ = 0.0038, p_ige, tha_ = 0.0050); while no age effect was observed in the control group in cortex and a significant increase was detected in the thalamus (NAA: p_age, tha_ = 0.0057). Moreover, in the cortex, the group effect was also significant in the concentration of these two metabolites (NAA: p_group, ctx_ = 0.0325; tNAA: p_group, ctx_ = 0.0370). The longitudinal evolution of cortical Cr and thalamic tCr were significantly affected by the genotype (NAA: p_group, ctx_ = 0.0325; tNAA: p_group, ctx_ = 0.0370), with a significant decrease with age in TgF344-AD rats (Cr: p_age, ctx_ = 0.0065; tCr: p_age, tha_ = 0.0040) and no effect (cortex) or significant increase (thalamus, tCr: p_age, tha_ = 0.0278) in WT. In the cortex, Tau and tCho concentrations were significantly affected by genotype (Tau: p_group, ctx_ = 0.0092; tCho: p_group, ctx_ = 0.0105) and showed significantly different temporal trajectories in TgF344-AD and WT animals (Tau: p_inter, ctx_ = 8.08·10^−5^; tCho: p_inter, ctx_ = 0.0445). Tau decrease with age was significant in the transgenic group (p_iage, ctx_ = 1.03·10^−5^), while no age effect was observed in WT animals. In the thalamus, Glu and Ins+Gly also showed significantly different age-dependent trajectories (Glu: p_inter, tha_ = 0.0138^5^; Ins+Gly: p_inter, tha_ = 0.0281). Glu significantly decreased in transgenic animals (p_age, tha_ = 0.0041), but not in controls while levels of Ins+Gly significantly increased in WT animals (p_age, tha_ = 0.0400), but not in TgF344-AD.

In the hippocampus, a significant interaction between age and group was detected for NAA (p_inter, hip_ = 0.0211), which significantly increased in the WT group (p_age, hip_ = 0.0171) but not in the transgenic group. In addition, a significant group effect was observed in tCho (p_group, hip_ = 0.0449), with increased values in TgF344-AD.

Finally, the striatum Glu concentration was significantly lower in transgenic animals (p_group, str_ = 0.0437). The age-dependent changes in Ins+Gly differed between groups (p_inter, str_ = 0.0178): although in both cases Ins+Gly concentration significantly increased with age (p_age, str_ = 7.95·10^−8^), the effect was much larger in the WT group (p_age, str_ = 0.0272).

Besides the evaluation of longitudinal trajectories, concentrations at specific time points were compared if a significant group or group/age interaction was found. Significant differences and η^2^ effect size are reported in Table 2.

**Table 2:** Between-group comparison of metabolite concentration at specific time points.

Region	METAB.	12 months		15 months		18 months	
		*p*-value	η^2^	*p*-value	η^2^	*p*-value	η^2^
Cortex	NAA	n.s	–	n.s	–	0.0176*	0.1579***
	Tau	n.s	–	0.0228*	0.3028***	0.0285*	0.2620***
Hippocampus	NAA	0.0095**	0.1166**	0.0095**	0.1752***	0.0284*	0.1121**
Striatum	Glu	n.s	–	0.0343*	0.2068***	n.s	–
Thalamus	tCr	0.0106*	0.1436***	0.0106*	0.1537***	0.0105*	0.0377*
	NAA	n.s	–	n.s	–	0.0105*	0.1052**
	Glu	n.s	–	0.0185*	0.2109***	n.s	–

Significant group differences between WT and transgenic animals at specific time points (only evaluated if statistically significant group-age interaction or group effect was observed in the LME model). *p*-value (* *p* < 0.05; ** *p* < 0.01; *** *p* < 0.001) and η^2^ effect size are reported. Asterisks represent small (*, η^2^ > 0.01), medium (**, η^2^ > 0.06) and large (***, η^2^ > 0.14) effects according to convention.

### Intra-regional coupling of longitudinal metabolic changes

**Figure 4:**
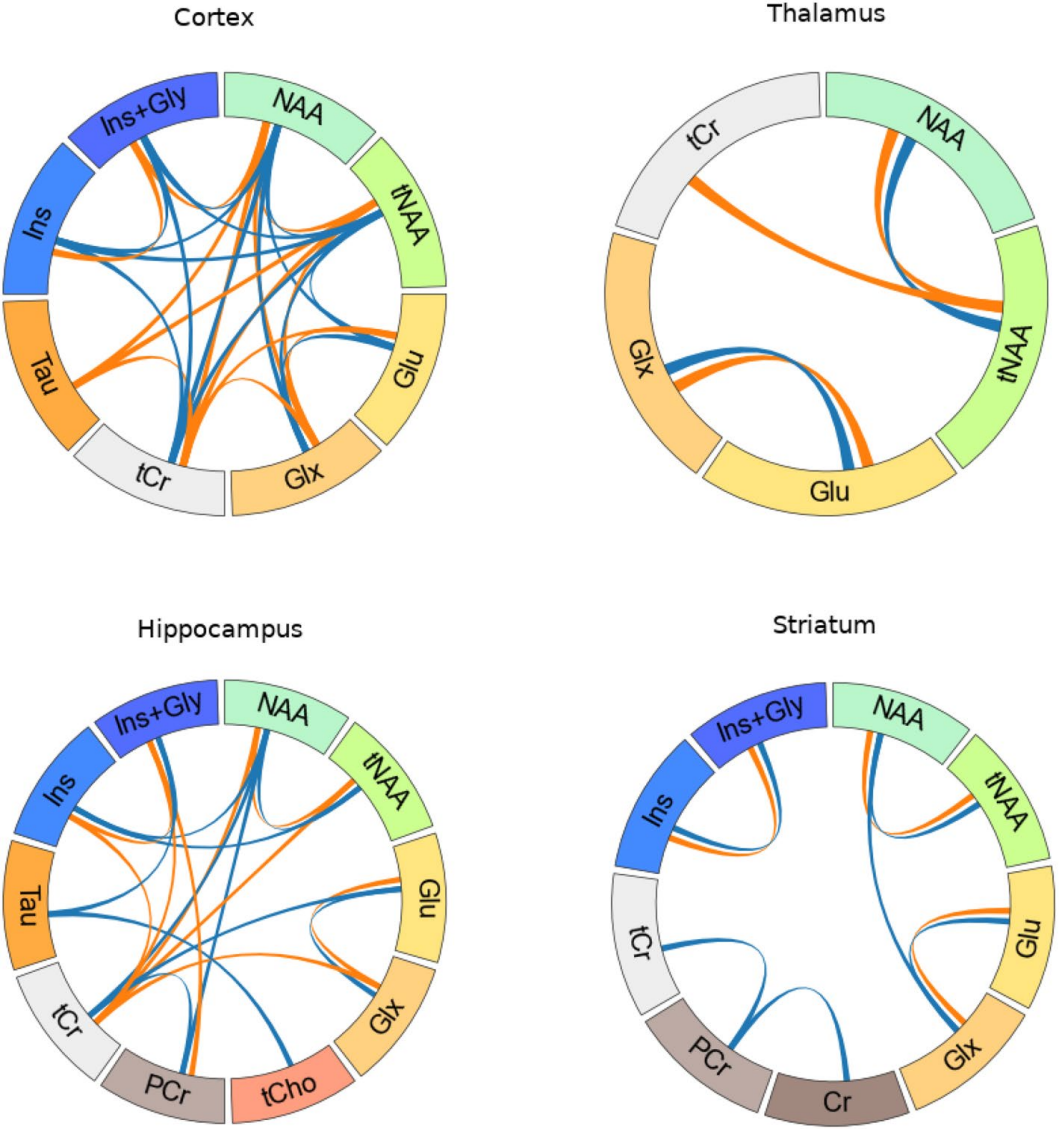
Intra-regional correlation. Correlation between the concentrations of metabolites within each region. Lines represent highly significant correlations (*p* < 0.001). Blue lines, correlation in WT rats; orange lines: correlation in TgF344-AD rats.

To evaluate specific metabolite interactions in the TgF344-AD rat model, we investigated the relationship between different metabolites within a specific region. This relationship was based on the correlation between the estimated concentration of each pair of metabolites taking into account all the time points. Figure 4 summarises the correlation between pairs of metabolites (only highly significant correlations are displayed, defined as *p* < 0.001). Correlation coefficients for every metabolite pair are reported in Supplementary Fig. 1.

While some metabolite correlation patterns were common to both groups, others were only observed in one of them. In cortex NAA, tNAA and tCr showed strong correlation with Tau (r_NAA, Tau_ = 0.75, r_tNAA, Tau_ = 0.71, r_tCr, Tau_ = 0.68) in TgF344-AD animals but with Ins and Ins+Gly (r_NAA, Ins_ = 0.73, r_NAA, Ins+Gly_ = 0.76, r_tNAA, Ins_ = 0.73, r_tNAA, Ins+Gly_ = 0.74, r_tCr, Ins_ = 0.82, r_tCr, Ins+Gly_ = 0.79) in the WT group. Also in the cortex, Glu and Glx correlated strongly with tCr in the transgenic group (r_Glu, tCr_ = 0.67, r_Glx, tCr_ = 0.69), but with NAA and tNAA in the WT animals (r_Glu, NAA_ = 0.66, r_Glu, tNAA_ = 0.69, r_Glx, NAA_ = 0.72, r_Glx, tNAA_ = 0.75).

Different correlation patterns were also observed in the hippocampus. Specifically, Ins correlated with tCr (r_Ins, tCr_ = 0.67) in TgF344-AD rats, but with NAA and tNAA (r_Ins, NAA_ = 0.65, r_Ins, tNAA_ = 0.67) in WT animals. In this region, the correlations between Ins+Gly and Tau (r_Ins+Gly, Tau_ = 0.8), Tau and tCho (r_Tau, tCho_ = 0.63), Glu and tCr (r_Glu, tCr_ = 0.64) and PCr and tCr (r_PCr, tCr_ = 0.72) were only significant in the WT group; while tCr was correlated with Ins, Glx and NAA (r_tCr, Ins_ = 0.67, r_tCr, Ins+Gly_ = 0.76, r_tCr, Glx_ = 0.69, r_tCr, NAA_ = 0.65, r_tCr, tNAA_ = 0.63) in the transgenic group.

In the striatum, the correlation between NAA and Glx was only significant in the WT group (r_NAA, Glx_ = 0.62). Likewise, PCr was strongly correlated with tCr and Cr only in the WT animals (r_PCr, tCr_ = 0.69, r_PCr, Cr_ = − 0.70). As reported in Supplementary Fig 1, while PCr and tCr correlation was positive, PCr and Cr were anticorrelated. In the thalamus, significant correlations were observed between NAA and tNAA (WT: r_NAA, tNAA_ = 0.74, TG: r_NAA, tNAA_ = 0.76) and between Glx and Glu (WT: r_Glu, Glx_ = 0.88, TG: r_Glu, Glx_ = 0.91) in both groups. Interestingly, TgF344-AD rats showed a significant correlation between Cr and tNAA (r_Cr, tNAA_ = 0.82) not observed in the WT animals.

### Inter-regional interaction of metabolites

**Figure 5:**
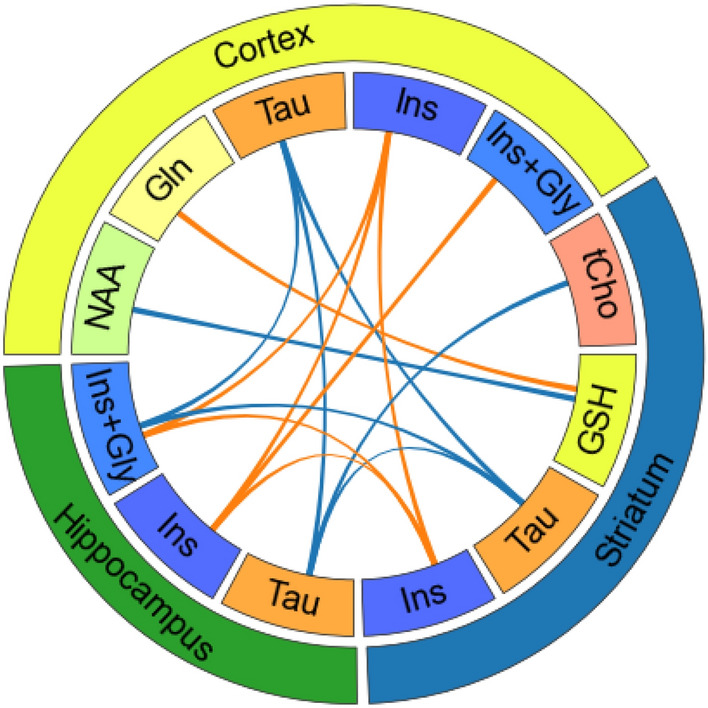
Inter-regional correlations. Correlation between metabolite concentrations in different brain regions. Lines represent highly significant correlations (*p* < 0.001). Blue, correlations in WT rats; orange, correlations in TgF344-AD rats. Intra-regional correlations between displayed metabolites, shown in Fig. 4, are not plotted here.

Finally, metabolic associations between different regions were also evaluated. The results are shown in Fig. 5. The inter-regional correlation coefficients between each metabolite/region pair are compiled in Supplementary Fig. 2.

The pattern of significant inter-regional correlations differed between WT and TgF344-AD rats. Namely, specific correlations across the hippocampus, striatum and cortex were observed for myo-inositol (either Ins or Ins+Gly) in the transgenic group (r_Ins hip, Ins ctx_ = 0.73, r_Ins hip, Ins+Gly ctx_ = 0.64, r_Ins+Gly hip, Ins ctx_ = 0.71, r_Ins+Gly hip, Ins+Gly ctx_ = 0.61, r_Ins hip, Ins str_ = 0.61, r_Ins+Gly hip, Ins str_ = 0.64, r_Ins ctx, Ins str_ = 0.70), but for Tau in the WT (r_Tau hip, Tau ctx_ = 0.81, r_Tau hip, Tau str_ = 0.65, r_Tau ctx, Tau str_ = 0.70). In this group, cortical and striatial Tau were also associated to hippocampal Ins+Gly levels (r_Tau ctx, Ins+Gly hip_ = 0.71, r_Tau str, Ins+Gly hip_ = 0.69); and hippocampal Tau was correlated to striatal tCho (r_Tau hip, tCho str_ = 0.61). Finally, while striatal GSH did not show significant differences between transgenic and WT animals, it correlated with cortical Gln in transgenic (r_GSH str, Gln ctx_ = 0.73) but with cortical NAA in WT animals (r_GSH str, NAA ctx_ = 0.73).

## Discussion

In this work, we aimed to characterise the spatio-temporal metabolic profile changes during the onset of AD-like pathology in the TgF344-AD rat brain. Longitudinal MRS studies were performed in the same cohort of WT and TgF344-AD animals. Four time points, from 9 to 18 months, were evaluated to capture the onset of metabolic changes: three points before the manifestation of gross pathological changes, as described in this model^27^; and another one later on. To account for specific regional differences in the progression of the disease, MRS data was acquired from four voxel positions, whose accurate location was evaluated based on automatic segmentation of structural MRI.

Based on a strict quality control and processing pipeline, the longitudinal metabolic profiles demonstrated progressive changes in TgF344-AD rats compared to WT, consistent with the major findings reported in clinical AD, such as decreased N-acetylaspartate and glutamate. Specifically, decreased NAA and/or tNAA in the cortex, hippocampus and thalamus, and decreased Glu in the thalamus and striatum were detected in TgF344-AD compared to WT animals, further confirming the suitability of this model for translational studies on AD progression. Additional analysis revealed strong intra-regional couplings of cortical NAA, tNAA and tCr with myo-inositol (either Ins or Ins+Gly) in WT, but with Tau in transgenics rats; whereas in the hippocampus, Tau showed strong correlations with Ins+Gly and tCho only in the WT group. Moreover, divergent inter-regional metabolic couplings in TgF344-AD and WT animals were observed. Specific crosstalks between cortex, hippocampus and striatum were found for Tau in the WT group and for myo-inositol in the TgF344-AD rats. This analysis sheds light into the complex spatio-temporal metabolic rewiring in AD, which would be linked to the pathological cascade associated to the disease^2,48^. The different associations of Ins and Tau with other metabolites in WT and TgF344-AD rats, suggest a relevant role for these two metabolites during the course of the pathology. While Ins has been related to neuroinflammation^4,49^ modulatory role in microglial activation has been associated to Tau^50^. This fact would be in line with the AD trajectory described in^48^, where it is suggested that Aβ deposition associated to ageing is followed by a protective microglial activation. Therefore, inappropiate microglial reponses leading to inflammatory response could be related with impaired coupling between Tau and Ins observed in the transgenic animals. The lack of effectivity in this early response would be followed by an increase in Aβ deposition, *tau* protein pathology and neurodegeneration, all of them associated with the decreased levels of NAA^4^ observed in our study, more evident at 15 and 18 months of age. Interestingly, NAA was coupled with Tau in transgenic animals but with Ins in wild-type animals, supporting this dual trajectory depending on the initial microglial response.

Importantly, the age-dependent decrease of cortical and thalamic creatine and its strong correlation with metabolites such as NAA or myo-inositol, reveals its inadequacy as an internal reference for relative quantification methods routinely used in the clinic (e.g. Ins/Cr), highlighting the need for careful interpretation of such results in the context of AD. To mention, the age-dependent evolution of creatine in hippocampus shown a similar pattern than in cortex and thalamus, although not significant differences were identified. Indeed, similar results were reported in a study with a bigger sample size including both male and female TgF344-AD, where significantly decreased levels of hippocampal tCr were observed in the transgenic group^33^.

Objective quality control was used to assure the reliability of the absolute metabolite concentrations estimated from each MRS dataset, including spectral selection based on SNR and FWHM, and fitting performance based on Cramér-Rao Lower Bounds assessment. Moreover, the study groups were assessed for potential outliers (based on interquartile range), controlled physiological condition throughout the acquisition (breathing and rectal temperature), and similar percentage of the region of interest covered by the MRS voxel. The slightly lower rectal and brain-estimated temperatures noticed in the cortex and thalamus of the transgenic group are consistent with previous findings in AD patients suggesting limited thermoregulation functions which have been associated with reduced number of suprachiasmatic nucleus neurotensin neurons^51,52^. Importantly, these differences, although significant, were not associated with the metabolic changes detected. It deserves to be mentioned that all the acquisitions were performed during the light period to avoid effects associated to the circadian cycle. It has been shown that diurnal variability in MRS measures is low and makes this technique reliable during this period^53^. Together with this, it has been shown that the use of isoflurane anaesthesia disrupts the differences associated to circadian phases in rats^54^ and therefore the results would not be significantly affected by the day-time of acquisition.

### ⁠Temporal metabolic profile changes in the TgF344-AD rat brain

Metabolic profile analysis showed different longitudinal evolutions of NAA and/or tNAA in the cortex, hippocampus and thalamus of WT and TgF344-AD rats. Specifically, these metabolites decreased during ageing only in transgenic animals. NAA is a marker of neuronal density and its decrease has been associated with neuronal loss or dysfunction^4^. Consistently, decreased NAA is one of the most reproducible findings in the cortex and hippocampus of AD patients^4^, as well as in rat^28,32,33^ and mouse models^15^. Progressive neuronal damage has been described in the TgF344-AD model^27^, aligned with decreased NAA also reported in the hippocampus and thalamus^32,33^. Since NAA/Cr has also been negatively correlated with Aβ burden^5,20^, decreased NAA at the latest time points in TgF344-AD animals could be also associated with the presence of Aβ plaques, as has been described at 16 months of age by Cohen et al.^27^ and verified by histopathological analysis in the study cohort at 18 months of age (Supplementary Fig. 3).

A decrease of Glu was observed in the thalamus of TgF344-AD rats during ageing, not observed in WT, while, in the striatum, TgF344-AD showed stable lower levels of Glu than WT rats over the study. Glu is one of the main neurotransmitters in the brain, essential for dendrite and synapse formation. Lower Glu concentration has been associated with neuronal loss and correlated with poor cognitive outcome^55^. Therefore, the observed changes are consistent with the neuronal loss described in the transgenic model^27^. Indeed, we observed significantly lower Glu levels at 15 months of age in TgF344-AD with respect to WT animals, when clear cognitive impairment has been described^27^. Glu neurotransmission is crucial for learning and memory function^56^, and therefore, the early lower levels of striatal Glu observed in transgenic rats could be related to the early learning deficits previously described in these rats , such as impairments in long-term spatial memory described at 4 months of age^33^, slower learning in a delay-non-match-to-sample test^44^ and impaired reversal learning on the Morris Water Maze^57^ and Barnes Maze^27^. In mouse models of AD, decreased Glu or Glu/Cr was found in the cortex and hippocampus^13,18,58,59^, correlating with Aβ plaque deposition^20^. In human cohorts, decreased cortical levels of Glx or Glu have been reported in AD, MCI and at-risk populations^4,55^, as well as non-significant decreases of Glx/Cr in the striatum and thalamus of AD patients^60^.

The time-course profiles of cortical Cr and thalamic tCr also showed an age-dependent decrease in TgF344-AD animals, not observed in the WT group. In the thalamus, significant lower tCr concentration was observed in transgenic with respect to WT animals at 12, 15 and 18 months of age. These changes are coherent with the decrease in tCr observed in hippocampus recently reported in the same model^33^ and might be associated with cell energetics disturbances occurring in neurodegenerative pathologies^61^, consistent with a neuroprotective effect of Cr against Aβ toxicity reported in the McGill-R-Thy1-APP rat model of AD^62^. Previous studies in AD or MCI patients reporting lower tCr levels in areas such as the hippocampus^63,64^ support our findings, probably related with a similar metabolism of tCr in human and other mammals as described in^65^, where it is suggested the relationship between PCr and glutamate neurotoxicity associated to AD as well as the role of oxidative stress (related to creatine metabolism) in neurodegenerative diseases. Indeed, oxidative stress has been described in the TgF344-AD animals, where decreased antioxidant capacity was reported in these animals^66^. In the same line, ex-vivo analysis revealed lower tCr levels in the hippocampus and frontal cortex in a transgenic mouse model of AD^67^. However, increased tCr levels were reported in the cortex of McGill-R-Thy1 rats^28^ and in the hippocampus and thalamic regions of TASTPM mice^22^. These two models showed early amyloid pathology but none of them replicate other aspects of the disease such as neurofibrillary tangles or widespread cell death^26,68^, that have been described in the TgF344-AD rats^27^. Therefore, discrepancies may be related to pathological processes associated to neurodegeneration or *tau* protein pathology^26^.

Importantly, these results warn that metabolite to tCr ratios should be analysed with caution, since tCr is not a constant reference in AD and therefore it can lead to conflicting conclusions.

Regarding myo-inositol, a metabolite commonly related to human AD^4^, the MRS literature typically quantifies it as mixed Ins+Gly levels, since the metabolic profiles of both metabolites significantly overlap. Thus, Ins and Gly were both included in our basis set for their individual quantification. Although Gly was not further evaluated after quality control assessment, it improved the estimations of Ins+Gly compared to Ins alone, as expected. Accordingly, we detected significant differences between the age-dependent trajectories of Ins+Gly levels in the thalamus and striatum of TgF344-AD and WT animals, characterized by their stronger increase with ageing in WT rats. Although myo-inositol has been considered a glial marker, with increased levels suggesting gliosis, its role in neuroinflammation or glial activation remains to be discerned^16,49^. In this line, while the TgF344-AD model presents early gliosis, already detected at 6 months of age^27^ and neuroinflammation^32,69^, we and others have not detected increased Ins in this model compared to WT^32^, and an increase in hippocampal Ins was only observed at advanced stages in^33^. Notwithstanding, striatal and thalamic median Ins+Gly levels tended to be higher in transgenic than in control animals at early stages, but lower at later ages (Fig. 3). These observations strengthen the importance of accounting for the timing of the disease when comparing literature findings, which might explain some of the discrepancies reported in human cohorts and animal models. For instance, even though Ins increases have been commonly reported in AD patients^4^, significant decreases of Ins/Cr have been also reported in the striatum, hippocampus and other regions in AD or dementia with Lewy’s body, compared to healthy controls^60^. In the APP/PS1 mouse, both decreased Ins/Cr^15^ and increased Ins/Cr were reported in the hippocampus^13,58^, while no changes were found in Ins absolute concentration in the cortex^18^. In the McGill-R-Thy1 rat model of AD, Ins decreased at 3 months of age in the hippocampus, while increased at 9 months of age in the hippocampus and 12 months of age in the frontal cortex^28^.

With regards to Tau, our results showed significant age-dependent decreases in the cortex of TgF344-AD rats, not observed in the WT group. Indeed, significant lower Tau concentrations were detected in the transgenic group with respect to WT at 15 and 18 months. These results are in line with previous findings as decreased Tau in the hippocampus of this model^33^, and decreased Tau/Cr ratio in AD patients^70^ or aged animal models^71^. However, a recent study with the TgF344-AD model depicted increased cortical Tau levels at 18 months of age^32^. This discrepancy may be related to differences in the MRS acquisition protocol (such as its ~ 70% longer echo time, leading to mixed J-coupling and T2 saturation effects, reflected in the detection and quantification of Tau), or different voxel location in the cortex, which in our study was more frontal. In this line, it has been shown that cortical amyloid deposition starts in frontal and temporal areas, affecting medial and posterior cingulate cortex only later on^34^. In fact, our results suggest a trend towards increased Tau levels in the cortex of transgenic animals before 12 months, but decreased levels at later time points, compared to controls. Thus, the differences could also be associated with regional specific timings of the pathological changes. Tau plays modulatory and regulatory roles in different physiological processes. It has been reported as a neuroprotector in neurodegenerative diseases such as AD and used to ameliorate several neurological disorders^50^. Indeed, Tau supplementation improved cognition in the APP/PS1 mouse model of AD^72^ and enhanced adult neurogenesis under both in vitro and in vivo conditions^73^.

Finally, cortical tCho revealed different age-dependent evolution in TgF344-AD rats compared to WT, showing (non-significant) increased level at earlier stages which decreased later on. The genotype had a significant effect in the hippocampus with increased tCho values in theTgF344-AD rats, although both groups followed a similar trajectory, in line with the results showed in this region in^33^. Accordingly, tCho age-effect differences between TgF344-AD and control rats in the hypothalamus and hippocampus were also reported in^32^. Choline compounds are found in myelin sheets and cell membranes and variations in tCho have been related to white matter integrity and membrane turnover, although opposite findings have been reported in the cortex of AD subjects^4^. Indeed, decreased tCho/Cr was detected in the prefrontal cortex and thalamus^60^, while increased levels were reported in the posterior cingulate cortex^74^. Moreover, increased tCho has been reported with ageing in both humans and animal models^49,75,76^, associated with demyelination, inflammation, and functional changes including cognitive decline^77^. As for myo-inositol, higher cortical tCho in young transgenic animals might suggest early damage, being demyelination and inflammation processes less evident at more advanced ages. Choline is liberated during cell membrane turnover, and therefore increases are related to neuronal degeneration, and has been observed in brain tumours, demyelinating disease or after traumatic brain injury^33,78^. On the other hand, decreased tCho at later time points could also be associated with neuronal damage and subsequent cognitive impairment, as described in aged TgF344AD rats^27^; also aligned with decreased hippocampal tCho observed in a drug-induced memory deficit model^79^. This strengthens again the importance of accounting for the timing of the disease in metabolic profile investigations.

### Intra-regional correlations of brain metabolic profiles

We further investigated the associations between different metabolites in each brain region, from a systems biology perspective. Thus, we found different metabolic correlation patterns in transgenic and wild-type groups.

As expected, there were significant correlations between NAA and tNAA, Glu and Glx and Ins and Ins+Gly, regardless of the brain region and group; except for Ins and Ins+Gly in the thalamus. The latter could be related to the significant age effect observed in this region for Ins+Gly, but undetected in Ins. Although Gly could not be evaluated independently in our study, its neuroprotective properties in neurodegeneration and memory impairment^80^ merit further investigation with more sensitive technique in the context of AD.

The strong correlations between creatine with other metabolites altered in TgF344-AD rats represent one of the main findings of this study. Although creatine has been commonly used as a reference in MRS analysis of AD, recent studies suggest the involvement of Cr in neuropathological mechanisms underlying dementia and neurodegeneration^33,63–65^, which would encourage to a careful interpretation of results based on ratios to Cr. Our results strengthen this rationale, since tCr is shown to be correlated with metabolites associated to pathological processes described in AD, such as neurodegeneration (NAA), neuroinflammation (Ins) or glutamate toxicity (Glu, Glx)^65^. In the cortex, tCr was highly correlated with NAA and tNAA in both TgF344-AD and WT groups, whereas correlations with Glu and Glx were specific to TgF344-AD, and with Ins+Gly and Ins were only found in WT animals. In the hippocampus, tCr correlated strongly with NAA in both groups; but with Ins, Ins+Gly and Glx only in TgF344-AD rats, and with Glu only in WT animals. Interestingly, in the thalamus and hippocampus, tCr only correlated with tNAA in the transgenic group. Altogether, these results suggest an important role for Cr in AD-like processes, since it was highly correlated with metabolites showing pathology specific time-course changes. This supports the idea that the commonly considered metabolic ratios to tCr, such as decreased NAA/tCr and increased Ins/tCr^4,67^, might not be appropriate in the case of AD-like pathologies, given the potential confounding effect of altered tCr levels.

Moreover, the correlations between taurine and myo-inositol with other metabolites also differed between TgF344-AD and WT animals. Several studies have suggested an important role of either Tau or Ins in AD pathology^4,15,18,28,33,58,60,70^, highlighting the need to better understand their changes and apparent discrepancies reported in the literature. Beyond concentration differences in specific regions, intra-regional metabolite correlation analysis provided new insights into this issue. Thus, cortical levels of Tau in TgF344-AD rats were highly correlated with NAA, tNAA and tCr, suggesting a link between Tau decreases and neuronal dysfunction; while in WT animals, cortical NAA, tNAA and Cr correlated with Ins+Gly and Ins. Due to the minimal sample size criteria defined, Tau concentration was not evaluated in the thalamus and, therefore, no correlations could be investigated in this case. Such metabolic correlation patterns could reflect compensatory effects between neuroinflammation (Ins)^4,49^ and neurotrophic (Tau)^50,72,73^ processes associated with normal ageing, potentially mitigated in transgenic animals. In TgF344-AD, impaired neurotrophic processes could be related to neuronal dysfunction, manifested by decreased NAA. This pathological mechanism would be in line with the AD trajectory proposed in^48^.

Further correlations were found in the hippocampus of WT animals, between myo-inositol and NAA and tNAA and between Tau and both Ins+Gly and tCho. While the metabolic interplay between Ins, tCho and Tau remains to be unravelled, these results suggest a role for Tau in the normal ageing process, similar to Ins and tCho in inflammation and degeneration during normal ageing, as described in humans and animal models^62–64^. Tau has a modulatory and regulatory function in physiological processes including modulation of neuroinflammation. Its neuroprotective and anti-neuroinflammatory role has been reported in neurodegenerative diseases^50^. The coupling between Tau and the two metabolites related with inflammation observed in wild type animals was lost in the transgenic animals (correlation coefficients were 0.24 between Tau and Ins+Gly and 0.09 between Tau and tCho), which might reflect a dysfunction associated to the lack of the modulatory effect of Tau to cope with neuroinflammation.

Highly significant (*p* < 0.001) correlations between Glu (or Glx) and NAA, tNAA and/or tCr were detected in the cortex and hippocampus, as well as between Glx and NAA in the striatum only WT rats (while correlation between Glx and NAA was 0.62 in WT animals, it decreases to 0.45 in transgenic rats). In this region, TgF344-AD rats showed lower levels of Glu compared to WT, which could partially be related with this decrease in the correlation values (indeed, the correlation between Glu and NAA decreases from 0.6 in WT to 0.4 in transgenic rats; while an increase is observed in the correlation coefficient between Gln and NAA: 0.11 in WT and 0.29 in transgenic). All this could potential reflecting Gln-Glu cycle changes, associated with the neuronal loss described in this model^27,32,65^.

### Inter-regional correlations of brain metabolic profiles

Finally, we investigated the correlation between metabolite concentration across different brain regions. In WT animals, Tau levels were strongly correlated between the cortex, striatum and hippocampus, and with Ins+Gly in the hippocampus. In turn, TgF344-AD rats exhibited the same inter-regional metabolic crosstalk between cortex, striatum and hippocampus for Ins, instead of Tau. This could be related to the specific correlations of cortical NAA (and tNAA) with Tau found in transgenic animals, but with Ins (or Ins+Gly) in the cortex and hippocampus of WT animals. Such metabolic couplings could reflect a whole brain imbalance in Tau levels associated with region-specific onsets of neuronal dysfunction^27,34,57^. Altogether, this would support the hypothesis of a balance between neuroinflammation (Ins) and neurotrophic and modulatory (Tau) processes during normal ageing impaired in TgF344-AD rats, what could be related with the hypothesis presented in^48^, suggesting that differences in microglial response to the deposition of β-amyloid are related with AD progression.

Interestingly, while no significant group or interaction effects were found for GSH or Gln, highly significant correlation of striatal GSH with cortical NAA in the WT group and with Gln in transgenic animals were observed. GSH is the most prevalent anti-oxidant in the brain^49,81^ and decreased levels have been reported in MCI and AD patients^81^ and in the APPTg2576 model of AD^18^. Decreased antioxidant capacity which has been previously reported in the TgF344-AD rats^66^ could be related with the genotype specific correlation between GSH and other metabolites. Assuming that the GSH cycle compensates for decreased excitatory neurotransmission during Glu-Gln shuttle inhibition^82^, the correlation between Gln and GSH in TgF4344-AD rats could be related to alterations in the normal Glu-Gln cycle, consistent with the decreased Glu levels observed in this group.

### Limitations

We acknowledge some limitations in our study. Firstly, the relatively small sample size. While only 18 animals were included, each one was evaluated at 4 time points, and consequently the fitting of the LME model included up to 72 samples. In addition, the rigorous criteria for LME analysis ensured enough significance and effect sizes, although some changes at specific ages might remain undetected. Since our study focused on the analysis of the longitudinal differences of brain metabolic profiles between control and transgenic animals, rather than differences at a specific age, this allowed us to increase the statistical power analysis, as well as being more sensitive to the timing of the pathologic changes. Due to the exploratory character of the study, we have not applied multiple comparison correction. Nevertheless, as shown in Supplementary tables 4 and 5, the effect size of the observed differences was large in most of the evaluated metabolites.

Some brain or cognitive alterations have been described after 18 months of age in the TgF344-AD rats. While the age-dependent pathological processes involved increased presence of Aβ plaques and stronger cognitive impairment shown at 26 months of age with respect to 18 month-old animals^27^, other alterations such as deficits in novel object recognition^27^and visual alterations have been reported only at 24 and 19 months of age respectively^83^.Therefore, another potential limitation could be the chosen endpoint of the study.

The cohort evaluated in this work belongs to a wider study, where the animals underwent cognitive evaluation every 3 months by a delayed non match to sample (DNMS) task as previosuly described^12^, and further research should be done to evaluate if the repetition of this task could have an impact in brain metabolism. In addition, only male animals were included in our experiments, and consequently we did not account for sex-related differences in the pathology. We acknowledge this is a limitation of our study, and female animals have been included in our ongoing investigations. To mention, a recent study including both female and male TgF344-AD reported limited effect of sex on MRS-based results in hippocampus, while a sex effect was identified in volumetric measures^33^.

Finally, as a general limitation of animal models of AD, they might not mimic all the changes occurring in patients. Although our findings are mostly consistent with metabolic changes previously described in AD patients, further research should ascertain the reproducibility of the novel results reported here for the first time, such as the intra- and inter-regional correlations.

## Conclusion

Beyond individual metabolite analysis in AD, our innovative analysis of the intra- and inter-regional metabolic cross-talks, provides new insights for the understanding of the metabolic processes underlying the pathology. Thus, TgF344-AD rats showed group-specific correlations in metabolites associated with neurodegeneration and neural dysfunction in the cortex and hippocampus, imbalances in taurine association with other metabolites, and strong couplings between the myo-inositol levels in different brain regions.

The choice of the brain region and time points of interest (age) represent key aspects when designing an MRS-based study of AD progression. Among the four structures evaluated, cortex and thalamus showed the strongest metabolic differences between transgenic and control animals. While metabolic alterations in cortex have been extensively reported in clinical databases, very few reports have evaluated metabolism in thalamus in human cohorts. Thus, our results support the need for further investigation of AD-related alterations in the thalamus, whose role in the disease has been recently highlighted by different studies. Moreover, certain metabolic differences might occur at a specific stage of the disease, such as Ins or tCho increases, and would condition the further evolution. These changes could be hindered if insufficient time points are included in the experimental design. Indeed, this could lead to inconsistent findings if attention is not paid to the timing of the disease and the specificities of each animal model. For this reason, our work focused on the longitudinal evolution of metabolic profile changes, from the prodromal stage, to characterize the disease progression in the TgF344-AD rat model.

In conclusion, we have demonstrated a complex spatio-temporal metabolic rewiring in the TgF344-AD rat model. The specific association in between Ins and Tau with other metabolites in WT and TgF344-AD groups, respectively, suggests an important role for these two metabolites and their imbalance during the course of the pathology. On the other hand, the age-dependent decrease of cortical Cr renders it into an inadequate reference for relative quantification methods routinely used in the clinic (e.g. Ins/Cr), highlighting the need for careful interpretation of such results in AD research.

## Supplementary Information

Supplementary Information 1.

Supplementary Information 2.

## Data Availability

The datasets used and/or analysed during the current study are available from the corresponding author on reasonable request.

## Abbreviations

AD: Alzheimer’s disease
Aβ: Amyloid-β
Cr: Creatine
CSF: Cerebrospinal fluid
FoV: Field of view
FWHM: Full width half-maximum
Gln: Glutamine
Glu: Glutamate
Glx: Glutamine and glutamate pool
GM: Grey matter
GSH: Glutathione
Ins: Myo-inositol
Ins+Gly: Myo-inositol and glycine pool
LME: Linear mixed effect
MCI: Mild cognitive impairment
MRI: Magnetic resonance imaging
MRS: Magnetic resonance spectroscopy
NAA: N-acetylaspartate
NAAG: N-acetylaspartylglutamate
PCr: Phosphocreatine
SNR: Signal to noise ratio
Tau: Taurine
tCho: Total choline compounds
tCr: Total creatine
tNAA: N-acetylaspartate and N-acetylaspartylglutamate
TE: Echo time
TPM: Tissue probability maps
TR: Repetition time
WM: White matter
WT: Wild-type

## References

[1] Dubois B, Hampel H, Feldman HH, Scheltens P, Aisen P, Andrieu S (2016). Preclinical Alzheimer’s disease: Definition, natural history, and diagnostic criteria. Alzheimer’s Dement..

[2] Jack CR, Bennett DA, Blennow K, Carrillo MC, Dunn B, Haeberlein SB (2018). NIA-AA research framework: Toward a biological definition of Alzheimer’s disease. Alzheimer’s Dement..

[3] Graff-Radford J, Kantarci K (2013). Magnetic resonance spectroscopy in Alzheimer’s disease. Neuropsychiatr. Dis. Treat..

[4] Wang H, Tan L, Wang HF, Liu Y, Yin RH, Wang WY (2015). Magnetic resonance spectroscopy in Alzheimer’s disease: Systematic review and meta-analysis. J. Alzheimer’s Dis..

[5] Murray ME, Przybelski SA, Lesnick TG, Liesinger AM, Spychalla A, Zhang B (2014). Early Alzheimer’s disease neuropathology detected by proton MR spectroscopy. J. Neurosci..

[6] Voevodskaya O, Sundgren PC, Strandberg O, Zetterberg H, Minthon L, Blennow K (2016). Myo-inositol changes precede amyloid pathology and relate to APOE genotype in Alzheimer disease. Neurology.

[7] Modrego PJ, Fayed N, Errea JM, Rios C, Pina MA, Sarasa M (2010). Memantine versus donepezil in mild to moderate Alzheimer’s disease : A randomized trial with magnetic resonance spectroscopy. Eur. J. Neurol..

[8] Marjańska M, Weigand SD, Preboske G, Wengenack TM, Chamberlain R, Curran GL (2014). Treatment effects in a transgenic mouse model of Alzheimer’s disease: A magnetic resonance spectroscopy study after passive immunization. Neuroscience.

[9] Do Carmo S, Cuello AC (2013). Modeling Alzheimer’s disease in transgenic rats. Mol. Neurodegener..

[10] Sabbagh JJ, Kinney JW, Cummings JL (2013). Alzheimer’s disease biomarkers in animal models: Closing the translational gap. Am. J. Neurdegener. Dis..

[11] Galeano P, Martino Adami PV, Do Carmo S, Blanco E, Rotondaro C, Capani F (2014). Longitudinal analysis of the behavioral phenotype in a novel transgenic rat model of early stages of Alzheimer’s disease. Front. Behav. Neurosci..

[12] Muñoz-Moreno E, Tudela R, López-Gil X, Soria G (2020). Brain connectivity during Alzheimer’s disease progression and its cognitive impact in a transgenic rat model. Netw. Neurosci..

[13] Chen SQ, Cai Q, Shen YY, Wang PJ, Teng GJ, Zhang W (2012). Age-related changes in brain metabolites and cognitive function in APP/PS1 transgenic mice. Behav. Brain Res..

[14] Liang S, Huang J, Liu W, Jin H, Li L, Zhang X (2017). Magnetic resonance spectroscopy analysis of neurochemical changes in the atrophic hippocampus of APP/PS1 transgenic mice. Behav. Brain Res..

[15] Oberg J, Spenger C, Wang FH, Andersson A, Westman E, Skoglund P (2008). Age related changes in brain metabolites observed by1H MRS in APP/PS1 mice. Neurobiol. Aging.

[16] Pardon M, Lopez MY, Yuchun D, Marjańs M, Prior M, Brignell C (2016). Magnetic resonance spectroscopy discriminates the response to microglial stimulation of wild type and Alzheime’s disease models. Sci. Rep..

[17] Trushina E, Mielke MM (2014). Recent advances in the application of metabolomics to Alzheimer’s disease. Biochim. Biophys. Acta.

[18] Dedeoglu A, Choi JK, Cormier K, Kowall NW, Jenkins BG (2004). Magnetic resonance spectroscopic analysis of Alzheimer’s disease mouse brain that express mutant human APP shows altered neurochemical profile. Brain Res..

[19] Jansen D, Zerbi V, Janssen CIF, Dederen PJWC, Mutsaers MPC, Hafkemeijer A (2013). A longitudinal study of cognition, proton MR spectroscopy and synaptic and neuronal pathology in aging wild-type and AβPPswe-PS1dE9 mice. PLoS ONE.

[20] Von KM, Basil K, Metzger F, Steiner G, Richards JG, Ozmen L (2005). Altered metabolic profile in the frontal cortex of PS2APP transgenic mice, monitored throughout their life span. Neurobiol. Dis..

[21] Yang D, Xie Z, Stephenson D, Morton D, Hicks CD, Brown TM (2011). Volumetric MRI and MRS provide sensitive measures of Alzheimer’s disease neuropathology in inducible Tau transgenic mice (rTg4510). Neuroimage.

[22] Forster D, Davies K, Williams S (2013). Magnetic resonance spectroscopy in vivo of neurochemicals in a transgenic model of Alzheimer’s disease: A longitudinal study of metabolites, relaxation time, and behavioral analysis in TASTPM and wild-type mice. Magn. Reson. Med..

[23] Xu W, Zhan Y, Huang W, Wang X, Zhang S (2010). Reduction of hippocampal N-acetyl aspartate level in aged APP Swe/PS1 dE9 transgenic mice is associated with degeneration of CA3 pyramidal neurons. J. Neurosci. Res..

[24] Drummond E, Wisniewski T (2017). Alzheimer’s disease: Experimental models and reality. Acta Neuropathol..

[25] Götz J, Bodea L, Goedert M (2018). Rodent models for Alzheimer disease. Nat. Rev. Neurosci..

[26] Leon WC, Canneva F, Partridge V, Allard S, Ferretti MT, DeWilde A (2010). A novel transgenic rat model with a full Alzheimer’s-like amyloid pathology displays pre-plaque intracellular amyloid-beta-associated cognitive impairment. J. Alzheimers Dis..

[27] Cohen RM, Rezai-Zadeh K, Weitz TM, Rentsendorj A, Gate D, Spivak I (2013). A transgenic Alzheimer rat with plaques, tau pathology, behavioral impairment, oligomeric Aβ and frank neuronal loss. J. Neurosci..

[28] Nilsen LH, Melø TM, Saether O, Witter MP, Sonnewald U (2012). Altered neurochemical profile in the McGill-R-Thy1-APP rat model of Alzheimer’s disease: A longitudinal in vivo 1 H MRS study. J. Neurochem..

[29] Anckaerts C, Blockx I, Summer P, Michael J, Hamaide J, Kreutzer C (2019). Early functional connectivity deficits and progressive microstructural alterations in the TgF344-AD rat model of Alzheimer’s disease: A longitudinal MRI study. Neurobiol. Dis..

[30] Smith LA, McMahon LL (2018). Deficits in synaptic function occur at medial perforant path-dentate granule cell synapses prior to Schaffer collateral-CA1 pyramidal cell synapses in the novel TgF344-Alzheimer’s disease rat model. Neurobiol. Dis..

[31] Pentkowski NS, Berkowitz LE, Thompson SM, Drake EN, Olguin CR, Clark BJ (2018). Anxiety-like behavior as an early endophenotype in the TgF344-AD rat model of Alzheimer’s disease. Neurobiol. Aging.

[32] Chaney AM, Lopez-Picon FR, Serrière S, Wang R, Bochicchio D, Webb SD (2021). Prodromal neuroinflammatory, cholinergic and metabolite dysfunction detected by PET and MRS in the TgF344-AD transgenic rat model of AD: A collaborative multi-modal study. Theranostics.

[33] Fowler CF, Goerzen D, Devenyi GA, Madularu D, Chakravarty MM, Near J (2022). Neurochemical and cognitive changes precede structural abnormalities in the TgF344-AD rat model. Brain Commun..

[34] Grothe MJ, Barthel H, Sepulcre J, Dyrba M, Sabri O, Teipel SJ (2017). In vivo staging of regional amyloid deposition. Neurology.

[35] Hanseeuw BJ, Betensky RA, Mormino EC, Schultz AP, Sepulcre J, Becker JA (2018). PET staging of amyloidosis using striatum. Alzheimer’s Dement..

[36] Hanseeuw BJ, Lopera F, Sperling RA, Norton DJ, Guzman-Velez E, Baena A (2019). Striatal amyloid is associated with tauopathy and memory decline in familial Alzheimer’s disease. Alzheimer’s Res. Ther..

[37] Aggleton JP, Pralus A, Nelson AJD, Hornberger M (2016). Thalamic pathology and memory loss in early Alzheimer ’ s disease : Moving the focus from the medial temporal lobe to Papez circuit. Brain.

[38] Van De Mortel LA, Thomas RM, Van Wingen GA (2021). Grey matter loss at different stages of cognitive decline: A role for the thalamus in developing Alzheimer’s Disease. J. Alzheimer’s Dis..

[39] Provencher SW (2001). Automatic quantitation of localized in vivo 1H spectra with LCModel. NMR Biomed..

[40] Avants BB, Epstein CL, Grossman M, Gee JC (2008). Symmetric diffeomorphic image registration with cross-correlation: Evaluating automated labeling of elderly and neurodegenerative brain. Med. Image Anal..

[41] Valdés-Hernández PA, Sumiyoshi A, Nonaka H, Haga R, Aubert-Vásquez E, Ogawa T (2011). An in vivo MRI template set for morphometry, tissue segmentation, and fMRI localization in rats. Front. Neuroinform..

[42] Ashburner J, Friston KJ (2005). Unified segmentation. Neuroimage.

[43] Schwarz AJ, Danckaert A, Reese T, Gozzi A, Paxinos G, Watson C (2006). A stereotaxic MRI template set for the rat brain with tissue class distribution maps and co-registered anatomical atlas: Application to pharmacological MRI. Neuroimage.

[44] Muñoz-Moreno E, Tudela R, López-Gil X, Soria G (2018). Early brain connectivity alterations and cognitive impairment in a rat model of Alzheimer’s disease. Alzheimers Res. Ther..

[45] Zhu M, Bashir A, Ackerman JJ, Yablonskiy DA (2008). Improved calibration technique for in vivo proton MRS thermometry for brain temperature measurement. Magn. Reson. Med..

[46] Oberg AL, Mahoney DW (2007). Linear mixed effects models. Methods Mol. Biol. Top. Biostat..

[47] Judd CM, Westfall J, Kenny DA (2017). Experiments with more than one random factor: Designs, analytic models, and statistical power. Annu. Rev. Psychol..

[48] Leng F, Edison P (2021). Neuroinflammation and microglial activation in Alzheimer disease: Where do we go from here?. Nat. Rev. Neurol..

[49] Chaney AM, Williams SR, Boutin H (2019). In vivo molecular imaging of neuroinflammation in Alzheimer’s disease. J. Neurochem..

[50] Jakaria M, Azam S, Haque ME, Jo SH, Uddin MS, Kim IS (2019). Taurine and its analogs in neurological disorders: Focus on therapeutic potential and molecular mechanisms. Redox Biol..

[51] Harper DG, Stopa EG, Kuo-Leblanc V, McKee AC, Asayama K, Volicer L (2008). Dorsomedial SCN neuronal subpopulations subserve different functions in human dementia. Brain.

[52] Colwell CS (2021). Defining circadian disruption in neurodegenerative disorders. J. Clin. Invest..

[53] Al-iedani O, Arm J, Ribbons K, Lea R, Lechner-Scott J, Ramadan S (2018). Diurnal stability and long-term repeatability of neurometabolites using single voxel 1H magnetic resonance spectroscopy. Eur. J. Radiol..

[54] Wren-Dail MA, Dauchy RT, Blask DE, Hill SM, Ooms TG, Dupepe LM (2017). Effect of isoflurane anesthesia on circadian metabolism and physiology in rats. Comp. Med..

[55] Conway ME (2020). Alzheimer’s disease: Targeting the glutamatergic system. Biogerontology.

[56] Ménard C, Gaudreau P, Quirion R, Kantak K, Wettstein J (2015). Signaling pathways relevant to cognition-enhancing drug targets. Cognitive Enhancement.

[57] Rorabaugh JM, Chalermpalanupap T, Botz-Zapp CA, Fu VM, Lembeck NA, Cohen RM (2017). Chemogenetic locus coeruleus activation restores reversal learning in a rat model of Alzheimer’s disease. Brain.

[58] Marjanska M, Curran GL, Wengenack TM, Henry P-GP, Bliss RL, Garwood M (2005). Monitoring disease progression in transgenic mouse models of Alzheimer’s disease with proton magnetic resonance spectroscopy. Proc. Natl. Acad. Sci..

[59] Mlynárik V, Cacquevel M, Sun-Reimer L, Janssens S, Cudalbu C, Lei H (2012). Proton and phosphorus magnetic resonance spectroscopy of a mouse model of Alzheimer’s disease. J. Alzheimer’s Dis..

[60] Su L, Blamire AM, Watson R, He J, Hayes L, O’Brien JT (2016). Whole-brain patterns of 1H-magnetic resonance spectroscopy imaging in Alzheimer’s disease and dementia with lewy bodies. Transl. Psychiatry.

[61] Rackayova V, Cudalbu C, Pouwels PJW, Braissant O (2017). Creatine in the central nervous system: From magnetic resonance spectroscopy to creatine deficiencies. Anal. Biochem..

[62] Brewer GJ, Wallimann TW (2000). Protective effect of the energy precursor creatine against toxicity of glutamate and beta-amyloid in rat hippocampal neurons. J. Neurochem..

[63] Tumati S, Martens S, Aleman A (2013). Neuroscience and biobehavioral reviews magnetic resonance spectroscopy in mild cognitive impairment : Systematic review and meta-analysis. Neurosci. Biobehav. Rev..

[64] Watanabe T, Shiino A, Akiguchi I (2008). Absolute quantification in proton magnetic resonance spectroscopy is superior to relative ratio to discriminate Alzheimer’s disease from Binswanger’s disease. Dement. Geriatr. Cogn. Disord..

[65] Wyss M, Kaddurah-Daouk R (2000). Creatine and creatinine metabolism. Physiol. Rev..

[66] Yang L, Wu C, Li Y, Dong Y, Wu CYC, Lee RHC (2022). Long-term exercise pre-training attenuates Alzheimer’s disease–related pathology in a transgenic rat model of Alzheimer’s disease. GeroScience.

[67] Salek RM, Xia J, Innes A, Sweatman BC, Adalbert R, Randle S (2010). A metabolomic study of the CRND8 transgenic mouse model of Alzheimer ’ s disease. Neurochem. Int..

[68] Howlett DR, Richardson JC, Austin A, Parsons AA, Bate ST, Davies DC (2004). Cognitive correlates of Aβ deposition in male and female mice bearing amyloid precursor protein and presenilin-1 mutant transgenes. Brain Res..

[69] Voorhees JR, Remy MT, Cintrón-Pérez CJ, El Rassi E, Khan MZ, Dutca LM (2017). (−)-P7C3-S243 protects a rat model of Alzheimer’s disease from neuropsychiatric deficits and neurodegeneration without altering amyloid deposition or reactive glia. Biol. Psychiatry.

[70] Zhang Y, Liu Z, Ji B, Liu L, Wu S, Liu X (2019). Metabolite profile of Alzheimer’s disease in the frontal cortex as analyzed by HRMAS 1H NMR. Front. Aging Neurosci..

[71] Zhu M, Akimana C, Wang E, Ng CK (2019). H-MRS quantitation of age-dependent taurine changes in mouse brain. Mol. Imaging Biol..

[72] Kim HY, Kim HV, Yoon JH, Kang BR, Cho SM, Lee S (2014). Taurine in drinking water recovers learning and memory in the adult APP/PS1 mouse model of Alzheimer’s disease. Sci. Rep..

[73] Kilb W, Fukuda A (2017). Taurine as an essential neuromodulator during perinatal cortical development. Front. Cell. Neurosci..

[74] Marjańska M, McCarten JR, Hodges JS, Hemmy LS, Terpstra M (2019). Distinctive neurochemistry in Alzheimer’s disease via 7T in vivo magnetic resonance spectroscopy. J. Alzheimer’s Dis..

[75] Harris JL, Yeh H-W, Swerdlow RH, Choi I-Y, Lee P, Brooks WM (2014). High-field proton magnetic resonance spectroscopy reveals metabolic effects of normal brain aging. Neurobiol. Aging.

[76] Duarte JMN, Do KQ, Gruetter R (2014). Longitudinal neurochemical modifications in the aging mouse brain measured in vivo by 1 H magnetic resonance spectroscopy. Neurobiol. Aging.

[77] Febo M, Foster TC (2016). Preclinical magnetic resonance imaging and spectroscopy studies of memory, aging, and cognitive decline. Front. Aging Neurosci..

[78] Stovell MG, Yan JL, Sleigh A, Mada MO, Carpenter TA, Hutchinson PJA (2017). Assessing metabolism and injury in acute human traumatic brain injury with magnetic resonance spectroscopy: Current and future applications. Front. Neurol..

[79] Woo D, Lenkinski RE (2014). Neurochemical changes observed by in vivo proton magnetic resonance spectroscopy in the mouse brain postadministration of scopolamine. Acad. Radiol..

[80] Ullah R, Jo MH, Riaz M, Alam SI, Saeed K, Ali W (2020). Glycine, the smallest amino acid, confers neuroprotection against d-galactose-induced neurodegeneration and memory impairment by regulating c-Jun N-terminal kinase in the mouse brain. J. Neuroinflammation.

[81] Chen JJ, Thiyagarajah M, Song J, Chen C, Herrmann N, Gallagher D (2022). Altered central and blood glutathione in Alzheimer’s disease and mild cognitive impairment: A meta-analysis. Alzheimer’s Res. Ther. Biomed. Central.

[82] Sedlak TW, Paul BD, Parker GM, Hester LD, Snowman AM, Taniguchi Y (2019). The glutathione cycle shapes synaptic glutamate activity. Proc. Natl. Acad. Sci. USA.

[83] Tsai Y, Lu B, Ljubimov AV, Girman S, Ross-Cisneros FN, Sadun AA (2014). Ocular changes in TGF344-AD rat model of Alzheimer’s disease. Investig. Ophthalmol. Vis. Sci..

