# Supplementary material for: Spatio-temporal metabolic rewiring in the brain of TgF344-AD rat model of Alzheimer’s disease
Source: Sci Rep. 2022 Oct 10;12:16958. doi: 10.1038/s41598-022-20962-6 (PMC9550832; doi:10.1038/s41598-022-20962-6)
Supplement: Supplementary file 1 — Supplementary Information 1. [file 41598_2022_20962_MOESM1_ESM.pdf]

SUPPLEMENTARY MATERIAL

Supplementary Table 1. Discarded spectra after quality control.

	Hippocampus		Thalamus		Striatum		Cortex	
	**WT**	**Tg**	**WT**	**Tg**	**WT**	**Tg**	**WT**	**Tg**
**9 months**	2	3	1	2	0	1	2	3
**12 months**	0	0	1	3	0	0	0	0
**15 months**	1	0	0	4	0	0	1	0
**18 months**	1	1	1	1	0	0	1	1

Number of spectra that were discarded because they do not complain with quality criteria (FWHM<=0.06 and SNR>10) by timepoint, region and group (WT: wild-type animals; Tg: transgenic TgF344-AD rats).

Supplementary Table 2. Metabolite concentration at each region and timepoint for wild-type and transgenic animals (see SupplementaryTable2.xlsx). Median and interquartile range are reported.

Supplementary Table 3. Additional quality criteria for magnetic resonance spectroscopy (MRS) acquisition.

	Hippocampus		Thalamus		Striatum		Cortex	
	**WT**	**Tg**	**WT**	**Tg**	**WT**	**Tg**	**WT**	**Tg**
Voxel location (%)	63.55±8.25	67.03±15.41	69.98±7.43	68.15±20.38	77.19±8.50	74.19±8.72	72.63±6.98	71.34±11.53
Brain temperature	36.23±1.01	35.89±1.01	35.89±0.84	35.55±1.02	35.55±1.12	34.88±1.35*	35.55±1.35	35.22±1.35*
Rectal temperature	35.30±0.98	34.84±0.93	35.42±1.12	34.73±0.98	35.43±1.10	34.70±0.91*	35.32±1.04	34.60±0.98*

Accuracy of the voxel location, estimated as the percentage of the MRS voxel covering the region of interest; and measured and estimated temperature during estimation were evaluated to ensure acquisition quality. Rectal measurement of temperature was performed during acquisition. Brain temperature was estimated based on chemical shift of the N-acetylaspartate (NAA) peak relative to water. Asterisks indicate significant differences between wildtype and transgenic animals.

Supplementary Table 4. Significant effects of age, group or age-group interaction in the metabolite concentration.

REGION	METAB.	Age		Group		Group x age interaction	
		p-value	Cohen's f^2	p-value	Cohen's f^2	p-value	Cohen's f^2
**Cortex**	**Cr**	0.0069**	0.6134***	n.s.	-	0.0408*	0.6245***
	**tCr**	0.0077**	0.3910***	n.s.	-	n.s.	-
	**NAA**	0.0060**	0.9723***	0.0325*	0.4156***	0.0027**	1.1117***
	**tNAA**	0.0072**	0.8372***	0.0370*	0.3457**	0.0049**	0.9624***
	**Glu**	0.0127*	0.2977**	n.s.	-	n.s.	-
	**Glx**	0.0267*	0.2726**	n.s.	-	n.s.	-
	**Tau**	1.09e-6***	2.4696***	0.0092**	0.9263***	8.08e-5***	2.5571***
	**tCho**	n.s.	-	0.0105*	0.2466**	0.0445*	0.7643**
**Thalamus**	**tCr**	0.0097**	1.7123***	n.s.	-	0.0012**	2.2174***
	**NAA**	0.0002***	2.0831***	n.s.	-	6.86e-6***	2.8519***
	**tNAA**	0.0119*	1.0185***	n.s.	-	0.0036**	1.4505***
	**Glu**	0.0028**	1.0504***	n.s.	-	0.0138*	1.1599***
	**Glx**	0.0356*	0.6520***	n.s.	-	n.s.	-
	**Ins+Gly**	n.s.	-	n.s.	-	0.0281*	0.6752***
	**tCho**	0.0031**	1.6175***	n.s.	-	n.s.	-
**Hippocampus**	**NAA**	n.s.	-	n.s	-	0.0211*	0.5779***
	**Ins**	0.0089**	0.3165**	n.s.	-	n.s.	-
	**Ins+Gly**	0.0116*	0.2298**	n.s.	-	n.s.	-
	**tCho**	n.s.	-	0.0449*	0.2116**	n.s.	-
**Striatum**	**Glu**	n.s.	-	0.0437*	0.3845***	n.s.	-
	**Ins**	0.0149*	0.8547***	n.s.	-	n.s.	-
	**Ins+Gly**	0.0395*	1.2619***	n.s.	-	0.0178*	0.7194***

Significant effects of age, group or age-group interaction in the metabolite concentration adjusted by linear mixed effects model (LME). p-value (* $p<0.05$; ** $p<0.01$; *** $p<0.001$) and Cohen's f^2 effect size are reported. Asterisks represent small (*, $f^2>0.02$), medium (**, $f^2>0.15$) and large (***, $f^2>0.35$) effect according to convention. Cr: creatine, tCr: total creatine; NAA: N-acetylaspartate; tNAA: NAA+N-acetylaspartylglutamate (NAAG) pool; Glu: glutamate; Glx: Glutamate and glutamine pool; Tau: taurine; tCho: choline compounds; Ins: myo-inositol; Ins + Gly: myo-inositol and glycine.

Supplementary Table 5. Significant age effects in the metabolite concentration in wild-type and TgF344-AD animals.

		CONTROL		TgF344-AD	
**REGION**	**METAB.**	**Age**		**Age**	
		**p-value**	**Cohen's f^2**	**p-value**	**Cohen's f^2**
**Cortex**	**Cr**	n.s.	-	0.0065**	0.3326**
	**NAA**	n.s.	-	0.0024**	0.3973***
	**tNAA**	n.s.	-	0.0038**	0.3780***
	**Tau**	n.s.	-	1.03e-5***	0.8274***
**Thalamus**	**tCr**	0.0278*	0.3443**	0.0040**	0.4784***
	**NAA**	0.0057**	0.3035**	0.0006***	0.7764***
	**tNAA**	n.s.	-	0.0050**	0.6552***
	**Glu**	n.s.	-	0.0041**	0.6062***
	**Ins+Gly**	0.0400*	0.1877**	s.s.	n.s.
**Hippocampus**	**NAA**	0.0171*	0.2690**	n.s.	-
**Striatum**	**Ins+Gly**	7.95e-8***	1.0284***	0.0272*	0.2374**

Significant effects of age detected after fitting linear mixed effect model to each group (estimated only if age-group interaction was significant in the whole-cohort model). p-value (* $p<0.05$; ** $p<0.01$; *** $p<0.001$) and Cohen's f^2 effect size are reported. Asterisks represent small (*, $f^2>0.02$), medium (**, $f^2>0.15$) and large (***, $f^2>0.35$) according to convention. Cr: creatine, tCr: total creatine; NAA: N-acetylaspartate; tNAA: NAA+N-a1cetylaspartylglutamate (NAAG) pool; Glu: glutamate; Tau: taurine; Ins + Gly: myo-inositol and glycine.

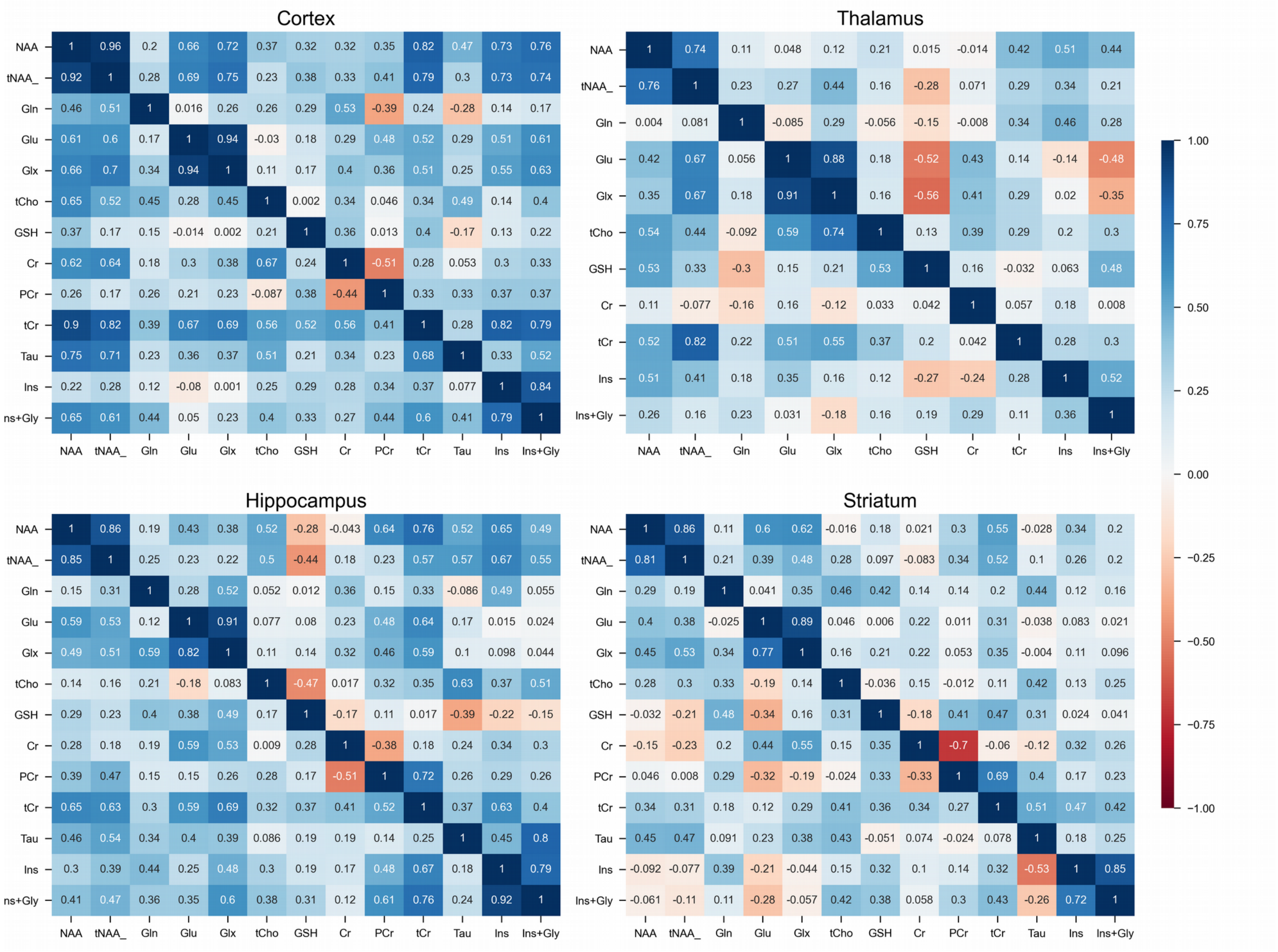

Supplementary Figure 1. Intra-regional correlation coefficients between metabolite concentration in each region. Upper triangle: correlation in wildtype animals; lower triangle: correlation in TgF344-AD. NAA: N-acetylaspartate; tNAA: NAA+N-acetylaspartylglutamate (NAAG) pool; Gln: glutamine; Glu: glutamate; Glx: Glu+Glx pool; GSH: glutathione; Cr: creatine; PCr: phosphocreatine; tCr: total creatine; Tau: taurine; Ins: myo-inositol; Ins + Gly: myo-inositol and glycine.

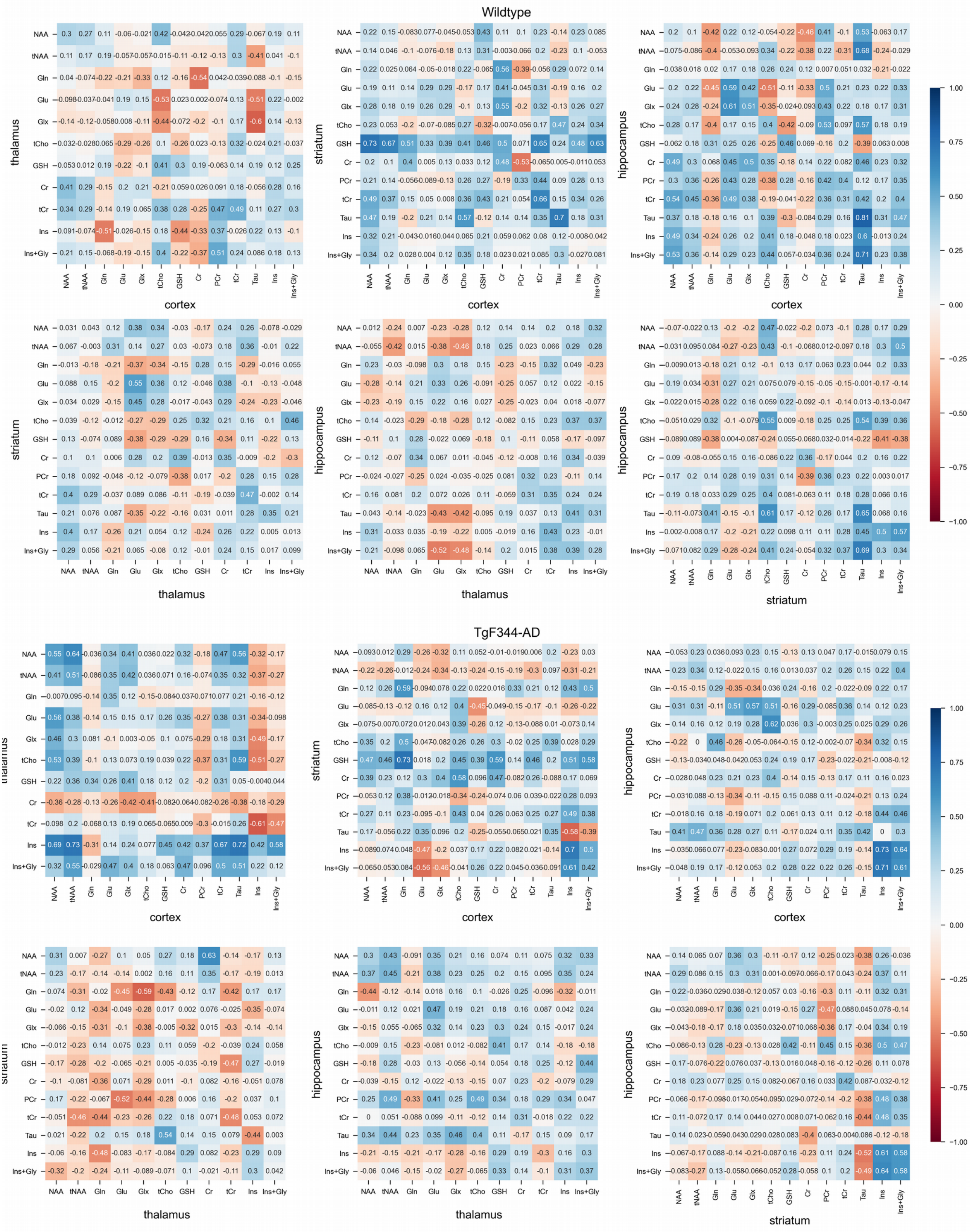

Supplementary Figure 2. Inter-regional correlation coefficients between metabolite concentration in each pair of regions in wildtype (superior rows) and TgF344-AD (inferior rows). NAA: N-Acetyl-Aspartate; tNAA: NAA+N-acetyl-aspartyl-glutamate (NAAG) pool; Gln: glutamine; Glu: glutamate; Glx: Glu+Glx pool; GSH: glutathione; Cr: creatine; PCr: phosphocreatine; tCr: total creatine; Tau: taurine; Ins: myoinositol; Ins + Gly: myoinositol and glycine.

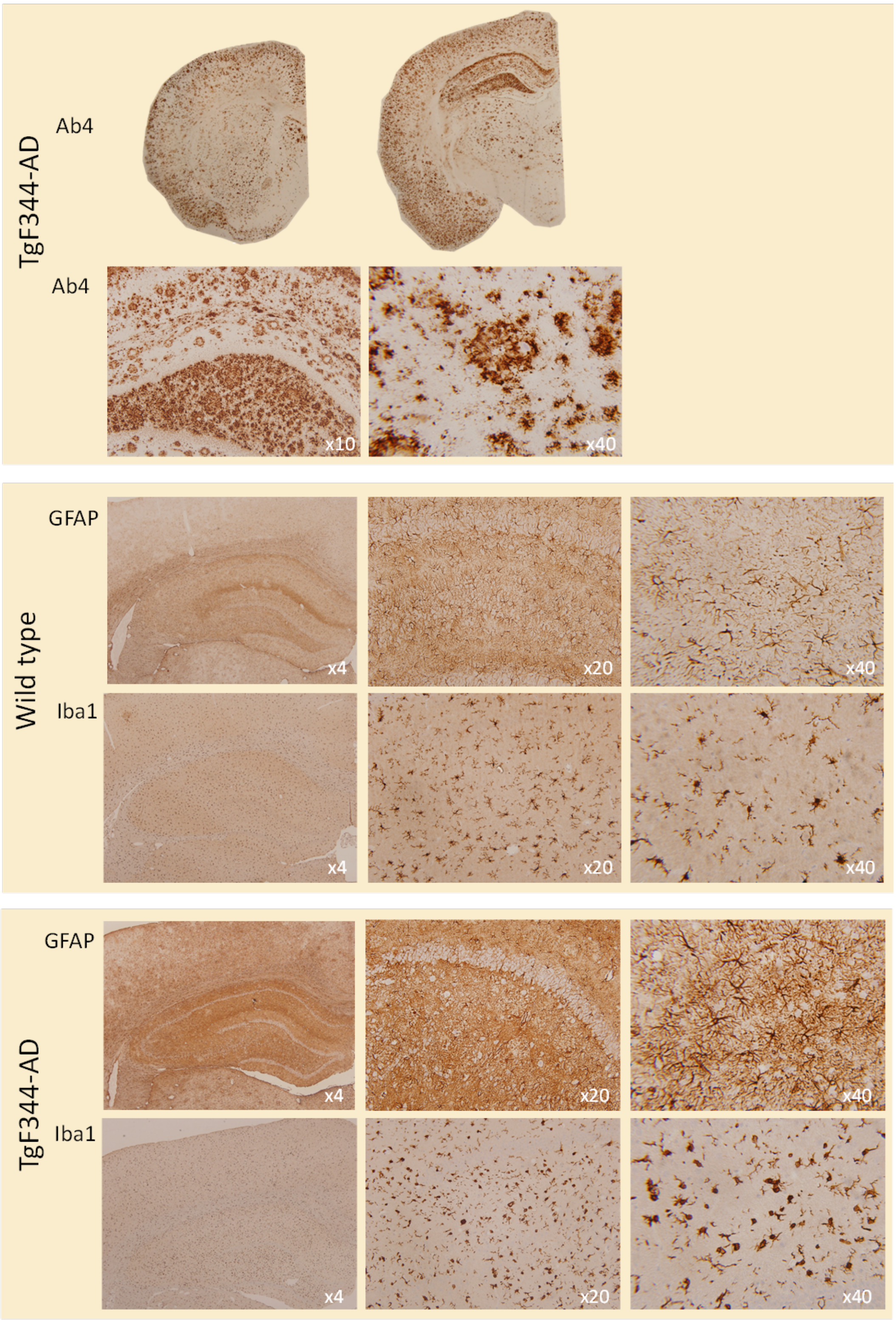

Supplementary Figure 3. Histological validation of β-amyloid plaques and neuroinflammation in TgF344-AD rats at 18 months of age. a) Representative coronal slices of TgF344-AD rat brain at the same levels where 1H MRS voxels were acquired (in example: striatum, cortex, hippocampus and thalamus). Magnifications at 10x are included showing the plaque load in the dentate gyrus of hippocampus and at 40x to reveal a classic b amyloid plaque. b) Representative astrocyte staining of the hippocampus in wild type animals at different magnifications. c) Representative microglia staining of the hippocampus in TgF344-AD rats at different magnifications. (beta4 antibody against Aβ, 1:50, Dako; GFAP antibody against astrocytes, 1:1000, Dako; Iba1 antibody against microglia, 1:400, Wako).
